# Heterogeneous Ion-Induced Nucleation of Water and Butanol Vapors Studied via Computational Quantum Chemistry beyond Prenucleation and Critical Cluster Sizes

**DOI:** 10.1021/acs.jpca.3c00066

**Published:** 2023-04-26

**Authors:** Antti Toropainen, Juha Kangasluoma, Hanna Vehkamäki, Jakub Kubečka

**Affiliations:** † University of Helsinki, Institute for Atmospheric and Earth System Research/Physics, Faculty of Science, P.O. Box 64, Helsinki 00140, Finland; ‡ Aarhus University, Department of Chemistry, Langelandsgade 140, Aarhus 8000, Denmark

## Abstract

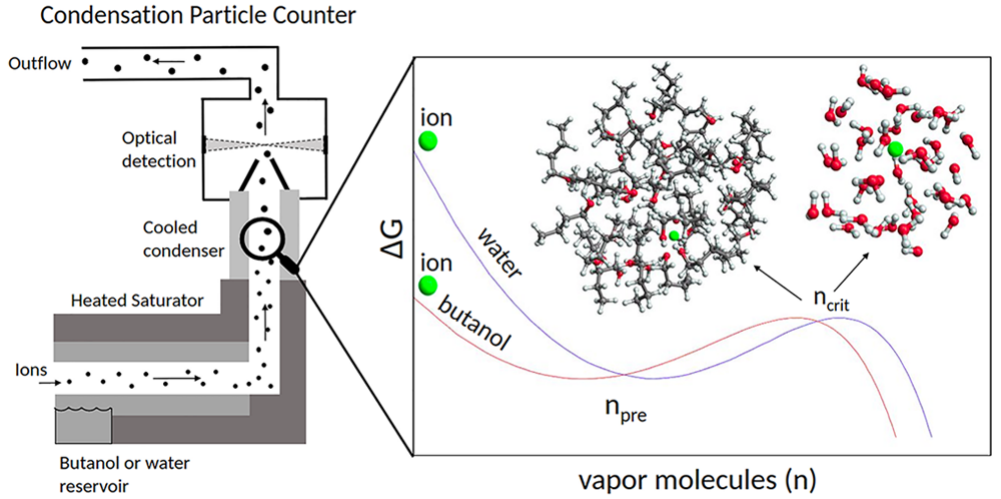

Water and butanol are used as working fluids in condensation particle counters, and condensation of a single vapor onto an ion can be used as a simple model system for the study of ion-induced nucleation in the atmosphere. Motivated by this, we examine heterogeneous nucleation of water (H_2_O) and *n*-butanol (BuOH) vapors onto three positively (Li^+^, Na^+^, K^+^) and three negatively charged (F^–^, Cl^–^, Br^–^) ions using classical nucleation theory and computational quantum chemistry methods. We study phenomena that cannot be captured by Kelvin–Thomson equation for small nucleation ion cores. Our quantum chemistry calculations reveal the molecular mechanism behind ion-induced nucleation for each studied system. Typically, ions become solvated from all sides after several vapor molecules condense onto the ion. However, we show that the clusters of water and large negatively charged ions (Cl^–^ and Br^–^) thermodynamically prefer the ion being migrated to the cluster surface. Although our methods generally do not show clear sign-preference for ion–water nucleation, we identified positive sign-preference for ion–butanol nucleation caused by the possibility to form stabilizing hydrogen bonds between butanol molecules condensed onto a positively charged ion. These bonds cannot form when butanol condenses onto a negatively charged ion. Therefore, we show that ion charge, its sign, as well as vapor properties have effects on the prenucleation and critical cluster/droplet sizes and also on the molecular mechanism of ion-induced nucleation.

## 1 Introduction

Phase transformation from gas to liquid is a central process in new particle formation (NPF), a mechanism forming approximately 50% of atmospheric aerosol particles.^1,2^ This process begins with the formation of a nucleation core and is followed by condensation of supersaturated vapors such as abundant water or low volatile organic compounds (e.g., oxidized products of α-pinene).^3^ The formation of these tiny particles is an example of a nucleation process and is hindered by an energy barrier by the costly construction of a new surface between gas and the newly formed liquid/solid particle. The lower the temperature, the lower the energy barrier. If the barrier is low enough (only a few kcal/mol at room temperature), it can be overcome by thermal fluctuations. Afterward, the formed particle spontaneously grows further.

The nucleation of a tiny new embryo in a metastable parent phase can occur homogeneously (i.e., only with a parent phase present) or heterogeneously onto a pre-existing surface.^4^ A special case of heterogeneous nucleation is ion-induced nucleation where the vapor condenses onto an atomic or molecular ion or a pre-existing charged particle. The electrostatic forces between the charged seed and vapor molecules enhance their mutual interaction, resulting in lower energy barrier compared to the neutral case. Ion-induced nucleation is one of several pathways through which atmospheric particles can form, and it has been shown to be of importance in the polar regions of the Earth^5^ and in the upper troposphere.^6^ Estimating the energy barrier of this process accurately is essential in predicting NPF rate in the atmosphere and the required critical supersaturation (i.e., the needed supersaturation to activate seed particles) in measurement instruments such as a condensation particle counter (CPC).

The energy barrier in atmospheric conditions (constant pressure and temperature) is described by the change in Gibbs free energy. In the framework of the classical nucleation theory (CNT), the fundamental tool for studying ion-induced nucleation is the classical Kelvin–Thomson (CKT) equation.^7^ The CKT assumes the formed ion-droplets are spherical in shape and possess the bulk properties (surface tension and dielectric constant) of the condensed liquid independent of droplet size. Due to these simplifications, the predictions made by the CKT theory regarding critical supersaturation, energy barrier, NPF rate, and electric mobility of the particles formed are often not in agreement with experimental studies.^8−12^ In addition, the CKT equation neither incorporates any charge sign preference nor takes into account the microphysical properties of the seed and/or the condensing vapor. Some deviations of experimental data from theoretical predictions have been speculated to be caused by the droplet geometry not being spherical or by the fact that small droplets (size of molecular clusters, i.e. several molecules) do not possess bulk liquid properties. In fact, it has been shown experimentally that a droplet needs to grow up to approximately 2 nm in radius for it to be satisfactorily described by the CKT theory.^13^ Hence, the CKT equation can hardly accurately capture ion-induced nucleation around tiny atomic ions (e.g., Li^+^).

Fangqun Yu (2005) suggested that the CKT equation may not account for the interaction between the core ion and the dipole moment of the vapor. He postulated a so-called modified Kelvin–Thomson (MKT) theory. Although MKT assumes the same bulk properties for the droplets as the CKT theory, Yu has indicated that MKT predicts some experimental results better than the CKT theory.^14^ However, it has also been reported that MKT might double count the dipole–dipole interaction,^15^ thus leading to too high binding energies.^16^ A subsequent study by Tauber et al.,^17^ who measured the activation of several charged monatomic ions by butanol, showed that neither CKT nor MKT theories can predict the required critical saturation ratio for seed activation, underlining the necessity of studying such systems with more accurate methods.

In this article, we provide molecular insight into ion-induced nucleation using quantum chemical (QC) calculations. We focus on ion-induced nucleation of water (H_2_O) and *n*-butanol (BuOH) condensing onto three positive (Li^+^, Na^+^, and K^+^) and three negative (F^–^, Cl^–^, and Br^–^) singly charged monatomic ions. These condensing vapors were selected as they are often used as working fluids in CPCs.^18^ Singly charged monatomic ions were chosen as they are the simplest type of ions. Several studies show that QC can be utilized to evaluate thermodynamic properties of charged molecular clusters.^19−24^ However, accurate QC calculations have a high computational cost which constrains their applicability to only small strongly binding molecular clusters. In our previous work,^25^ we showed that configurational sampling (CS) only up to a semiempirical level of theory is computationally effective even for large molecular clusters compared to the density functional theory (DFT) or other QC level schemes. Additionally, this method was found to produce similar cluster geometries and qualitatively similar free energy trends compared to those obtained with a combination of density functional theory (DFT, LC-ωHPBE/def2TZVP) with single-point electronic energy at a coupled cluster level (DLPNO–CCSD(T)/aug-cc-pVTZ).^25^ Therefore, in this work, we use this semiempirical level of theory to examine clusters containing even several tens of molecules, which allows us to reveal the molecular mechanism behind the ion-induced nucleation of the studied systems.

## 2 Theory behind Nucleation

### 2.1 Classical Nucleation Theory

In the case of a single-component homogeneous nucleation, CNT assumes transformation of a gas into a spherical liquid droplet. At atmospheric conditions (constant temperature and pressure), this process is characterized by Gibbs free energy change
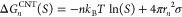
1where σ is the surface tension of the droplet, *S* is the saturation ratio of the vapor defined as a ratio between the actual and saturation vapor pressures (*S* = *p*_vap_/*p*_sat_), and *r*_*n*_ is the radius of a droplet with *n* condensed molecules. The first term accounts for the gain in free energy due to phase transformation from supersaturated vapor to liquid (i.e., *S* > 1 during nucleation), whereas the second term represents the work required for the formation of the new surface.

The cluster radius can be approximately estimated from pure bulk liquid properties (density, ρ, or molecular volume, *V*_m_)
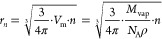
2where *N*_A_ is the Avogadro constant and *M*_vap_ is the molecular mass of vapor. The Gibbs free energy profile determined by eq 1 has only a single barrier, the top of which is located at critical cluster size (*r*_n_^*^)
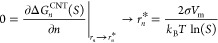
3The above equation is the well-known Kelvin equation.^26^

We have here expressed Δ*G*_*n*_^CNT^ in terms of number of molecules, but note that CNT is defined for continuous droplet size and no division to discrete molecules is assumed (i.e., *n* ∈ ⟨0, *∞*)).

### 2.2 Ion-Induced Nucleation

When an ion seed is present in the nucleating vapor, eq 1 has to be modified to account for the electrostatic potential. This gives rise to the classical Kelvin–Thomson (CKT) equation
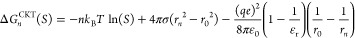
4where *q* is the number of elementary charges (*e*) carried by the ion, ε_0_ and ε_r_ are the vacuum permittivity and the relative permittivity of the condensed liquid, respectively, and *r*_0_ and *r*_*n*_ are the ion and droplet radii, respectively. The last term represents the change in electrostatic potential energy when adding vapor molecules onto the ion seed. The droplet radius can be calculated similarly as in the liquid-drop approximation (eq 2), this time taking into account the ion radius
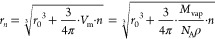
5

Similarly as in eq 3, the extremes in ion-induced free-energy profile can be found by combining eqs 4 and 5 and searching for the points where the derivative of Δ*G*_*n*_^CKT^(*S*) becomes zero, leading to
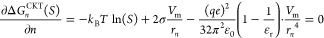
6The above equation can have up to two analytical solutions with a physical meaning. However, we do not present their complex formulas here. The first solution (with smaller *n*) we hereafter refer to as to the prenucleation droplet size (*n*_pre_), and it corresponds to the minimum on the Δ*G*_*n*_^CKT^ energy profile. The minimum of the free-energy profile is a consequence of the Coulombic interaction between the core ion and polarized vapor molecules. This interaction is so strong that it overpowers the cost of creating a new surface (second term in eq 4), and therefore Δ*G*_*n*_^CKT^ decreases until the prenucleation cluster size has been reached. Upon surpassing the prenucleation cluster, Δ*G*_*n*_^CKT^ grows until critical droplet size (*n*_crit_) is reached. This is the second solution (with larger *n*) to eq 6 and corresponds to the maximum of the Δ*G*_*n*_^CKT^ energy profile. At the Δ*G*_*n*_^CKT^ maximum, the free energy gain due to phase transition from supersaturated vapor to liquid (first term in eq 4) equals the cost of new surface formation. The difference between the minimum and the maximum defines the height of the nucleation barrier
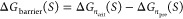
7The height of the energy barrier is dictated, besides other factors, by the level of saturation of the condensing vapor. Experimentalists often search for the onset saturation ratio (*S*_onset_) that corresponds to such a vapor pressure at which the nucleation (activation) probability of an ion seed within the experimental setup corresponds to 50%. The onset saturation ratio is of interest as around this value the nucleation probability drastically increases with vapor concentration. Only for the purposes of this article, we define onset saturation ratio *S*_onset_ so that the energy barrier equals 5 kcal/mol (i.e., Δ*G*_barrier_(*S*_onset_) = 5 kcal/mol). This energy barrier is surmountable by thermal fluctuation, but its true connection with actual experimental onset saturation ratio is strongly dependent on the instrument setup. Once the experimental setup is known, one could use, e.g., Atmospheric Cluster Dynamics Code (ACDC)^27^ to model the activation probability. In this work, a fixed onset barrier is selected so that the free-energy profiles for different systems can be compared to each other.

The ion and vapor properties used in this study are summarized in Tables 1 and 2. The values of ion radius were obtained from experimental interatomic distance measurements^28^ which were subsequently revised.^29^

**Table 1:** Effective Ion Radii for All Studied Ions^29^

	*r*_0_ [pm]		*r*_0_ [pm]
Li^+^	90	F^–^	119
Na^+^	116	Cl^–^	167
K^+^	152	Br^–^	182

**Table 2:** Bulk Liquid Properties of the Nucleating Vapor^a^

	ρ [kg·m^–3^]	σ [N/m]	ε_r_	*p*_sat_ [Pa]
water	997^30^	0.07199^31^	78.304^32^	3169.9^30^
butanol	810^33^	0.024204^34^	17.3^35^	905^36^

a Density (ρ), surface tension (σ), relative permittivity (ε_r_), and saturation vapor pressure (*p*_sat_) at 298.15 K.

**Figure 1:**
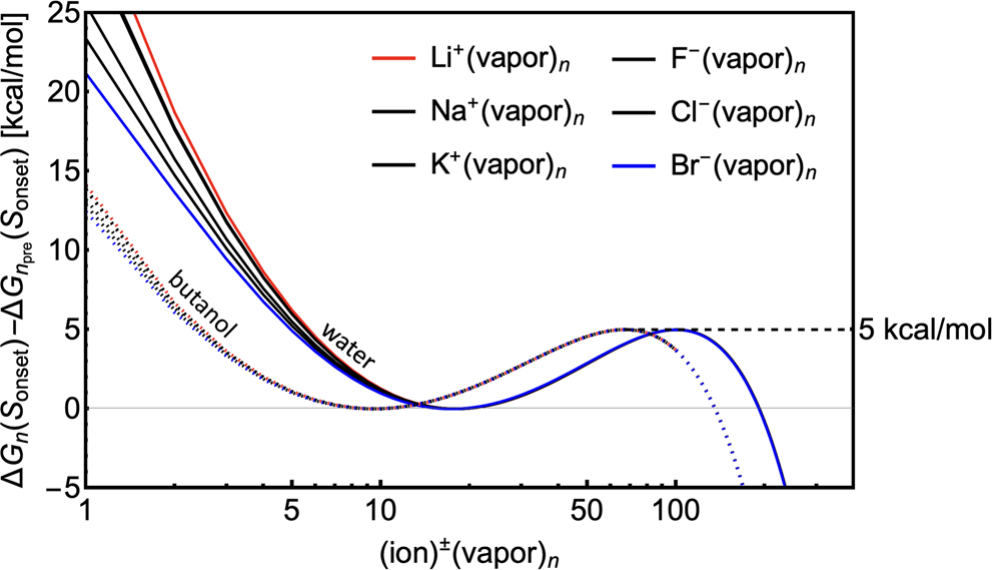
Gibbs free energy profile for ion-induced nucleation using classical Kelvin–Thomson (CKT) theory (eq 4). For each nucleating vapor, the onset saturation ratio is chosen so that the energy barrier is 5 kcal/mol.

Figure 1 shows the CKT-based Gibbs free energy profiles of ion-induced nucleation at room temperature (*T* = 298.15 K) for all studied systems. As we are using the CKT theory, the sign of the charged ion has no effect. Additionally, the properties of the ions have only a minor effect on the free energy profiles at small droplet sizes. Therefore, only the properties and concentrations of nucleating vapor affect the sizes of prenucleation and critical clusters. By using eqs 6 and 7, we calculated *S*_onset_ to be 3.425 and 2.910 for water and butanol, respectively, and regardless of ion, we get *n*_pre_ ≈ 18 and *n*_crit_ ≈ 100 for water and *n*_pre_ ≈ 9 and *n*_crit_ ≈ 67 for butanol.

**Figure 2:**
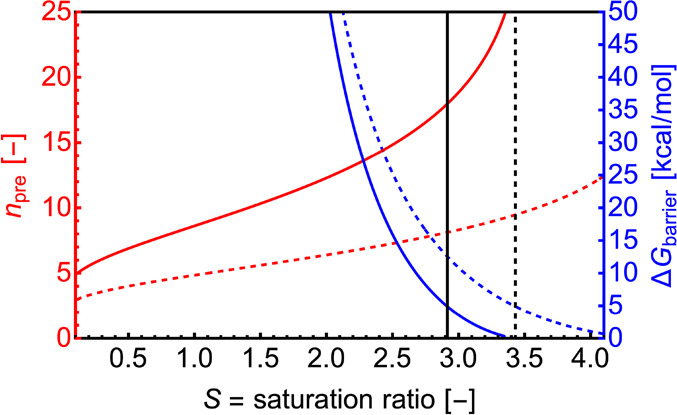
Dependency of prenucleation droplet size (*n*_pre_; red) and energy barrier height (Δ*G*_barrier_; blue) on saturation ratio *S* based on CKT for both vapors: water (solid line) and butanol (dashed line). The onset saturation ratios corresponding to a barrier of 5 kcal/mol are highlighted by solid and dashed vertical lines for water and butanol, respectively.

To make the connection between the studied properties more clear, we discuss the dependency (derived from CKT) of the free-energy barrier Δ*G*_barrier_ and size of prenucleation cluster *n*_pre_ on saturation ratio *S*. Figure 2 shows that the energy barrier rapidly decreases with saturation ratio. The process actually becomes barrierless and purely kinetically controlled with a high enough saturation. For *S* ≤ 1, the energy barrier is infinitely high, i.e., nucleation does not occur. In the figure, the crossings of black vertical lines and blue lines represent the saturation ratios (different for the two vapors) at which the energy barrier equals the desired 5 kcal/mol. As the saturation ratio increases, the core ion is able to accommodate a greater number of vapor molecules and the prenucleation cluster size grows. Additionally, a minimum of free-energy profile, which corresponds to *n*_pre_, also exists for *S* ≤ 1, where no nucleation occurs. Interestingly, a prenucleation cluster exists even at *S* = 0 due to strong Coulomb interaction between the ion and vapor molecules. In the figure, the crossings of black vertical lines and red lines correspond to prenucleation sizes at our onset conditions.

### 2.3 Molecular View into Ion-Induced Nucleation

In this work, we utilize computational chemistry to analyze the first steps of ion-induced nucleation from the molecular point of view. Hence, we calculated thermodynamic properties of ion^±^(vapor)_*n*_ clusters using quantum chemistry (QC) methods (details are discussed in section 3). Each chemical species is thus characterized by Gibbs free energy (*G*) at reference conditions. *G* is calculated from the enthalpy (*H*) and entropy (*S*) or, as defined in the Gaussian program, from the electronic energy (*E*_el_) and thermal contributions (*dg*):
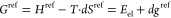
8Typically, the calculations are performed at standard conditions, i.e., the temperature of 298.15 K and atmospheric pressure *p*_ref_ = 1 atm. The condensation of vapor molecules onto an ion is characterized by a free energy of cluster formation (Δ*G*)
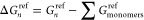
9

In one-component nucleation (e.g., single vapor), the free energy profile at a given actual vapor pressure Δ*G*_*n*_^QC^(*S*) (often called the actual Gibbs free energy) can be calculated as
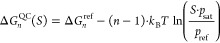
10where *S* = *p*_vap_/*p*_sat_. Equation 10 corresponds to CKT eq 4,^37^ and we can thus compare if the positions of prenucleation and critical clusters predicted by QC differ from prenucleation and critical droplet sizes given by CKT when the free-energy barrier heights are equal.

## 3 Methodology

### 3.1 Computational Chemistry

To the best of our knowledge, no research group has yet studied heterogeneous nucleation of BuOH on various ions via using QC. One reason why we also study the nucleation of water (H_2_O) vapor is to validate our methodology by comparing it to previous computational studies.^38−44^ In our previous work,^25^ we studied the condensation of butanol on a salt seed formed of Na^+^ and Cl^–^ ions. We showed that a tight-binding semiempirical method implemented in the XTB program^45,46^ qualitatively correlates with high-level QC calculations. The GFN1-xTB (from now on abbreviated as XTB) method is commonly used in configurational sampling procedures for molecular clusters. Due to high speed, endurance to fail/crash, and accuracy better than that of many other semiempirical methods (e.g., PM6), we use the XTB method for CS also in this work. For final structure optimization and energy evaluation, we chose the ωB97-XD/6-31++G(d,p)^47^ method implemented in the Gaussian 16 program,^48^ since it has been successfully used in many other studies of molecular clusters,^37,49−51^ and it provides good geometries and sufficiently accurate vibrational frequencies.^49,52^ ωB97-XD/6-31++G(d,p) has also been shown to provide the best estimate of binding energies when combined with a higher level single point electronic energy method (e.g., DLPNO–CCSD(T)).^53,54^ In this work, we only focus on the principles of the molecular mechanism and qualitative behavior of free-energy nucleation profiles, and therefore only the XTB and ωB97-XD/6-31++G(d,p) (from now on abbreviated as DFT) methods are used, although we note that using a larger basis set would also lead to more accurate binding energies. Likewise we note that the formation free energy of molecular clusters can also be evaluated with molecular dynamics (MD) simulations, as has been done, for example, by Tanaka et al. and Angélil et al.^55,56^ Although we will study nucleation with computational quantum chemistry focusing on minimum free energy structures in this work, performing MD simulations for our (ion)^±^(vapor)_*n*_ system could bring additional insight into dynamical behavior of the ionic clusters.

For each ion^±^(vapor)_*n*_ cluster, we performed configurational sampling (CS) with methodologies described by Kubečka et al.^51^ to obtain the lowest Gibbs free energy minimum structures. The CS was performed with the newest version of the ABCluster program (version 3.0) which couples the potential energy surface (PES) exploration of rigid molecules, the conformational exploration (variation in torsion angles within single molecules, which is only relevant in the case of the butanol molecule), and the subsequent semiempirical optimization step.^57,58^ Hence, the vapor molecule (H_2_O and BuOH) structures were treated as flexible building blocks at this stage (as opposed to the traditional rigid building blocks used in many previous studies^51^). The PES explorations have been performed via the artificial bee colony (ABC^59^) algorithm implemented in the ABCluster program^57,58^ with the parameters described in Table 3. The table shows the maximum minima explored which accounts for all independent repetitions of the ABCluster simulations with a given number of generations. The quality of CS is low for large clusters as is discussed in the next section. The energy evaluation and subsequent optimization of structures was done at the XTB level of theory.

**Table 3:** Summary of ABCluster^57,58^ Parametrizations^a^

cluster	generations	repetitions	minima explored
ion^±^(vapor)_1–10_	1000	3	3000
ion^±^(vapor)_11–20_	500	6	3000
ion^±^(vapor)_21–40_	50	3	150
ion^±^(H_2_O)_10·(5–15)_	50	3	150

a “ion^±^” stands for all ions: Li^+^, Na^+^, K^+^, F^–^, Cl^–^, and Br^–^. “vapor” stands for both nucleating vapors: H_2_O and BuOH.

In the next step, we have identified redundant (identical and energetically high-lying) structures, which were filtered out. From the remaining data set, we selected a representative set of 100 structures using a uniform sampling from a planar space defined by normalized energies and gyration radii of all configurations. For the selected sets, we performed vibrational analysis at the XTB level of theory to obtain room-temperature (*T* = 298.15 K) Gibbs free energies.

Finally, we selected the 20 energetically lowest-lying structures from the previous step and optimized them at the DFT level. Note that we selected only a small number of configurations mainly due to the computational cost. Although this is enough for qualitative understanding of the mechanism behind nucleation, one should be aware that quantitative calculations (e.g., cluster growth rate and particle formation rate) would require a more thorough CS.

### 3.2 QC Data Extrapolation

Since DFT calculations have significantly greater computational cost compared to the XTB calculations (especially for large systems), we only performed DFT calculations for the ion^±^(H_2_O)_0–15_ and ion^±^(BuOH)_0–8_ clusters out of all the clusters shown in Table 3. These results were used to calculate thermodynamic properties, i.e., Gibbs free energies of the clusters. Since semiempirical calculations are computationally cheap, we use them to extrapolate the DFT free energies toward large clusters. Here, as the first approximation, we assume that the difference between DFT and XTB has a linear dependence on cluster size (see SI-1 for reasoning behind this approximation)
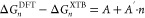
11After fitting the *A* and *A*′ parameters in eq 11 on small clusters, we can extrapolate DFT free energies using XTB data at standard conditions (i.e., *T* = 298.15 K and *p*_ref_ = 1 atm).

We, as most other studies, take into account only the global free-energy minima (Δ*G*_*n*,GM_). Hence, the conformational entropy contribution from other minima is neglected, which certainly introduces inaccuracy in these liquid-like systems. The contribution from other local minima can be approximately calculated by accounting for superposition over all energetically low-lying minima (Δ*G*_*n*,*i*_)^51^
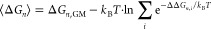
12where ΔΔ*G*_*n*,*i*_ = Δ*G*_*n*,*i*_ – Δ*G*_*n*,GM_. Accounting for other minima lowers the cluster Gibbs free energy and thus stabilizes the clusters. The greater the number of low-lying minima, the greater the cluster stability. The number of minima on the PES grows exponentially with cluster size,^60^ and therefore identifying all minima is impossible. To estimate the effect of multiple minima, we introduce another approximation, where the density of energy states has proportionally the same distribution  regardless of cluster size but the number of states scales exponentially
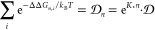
13where *K* is just a scaling factor. Hence eq 12 can be expressed as
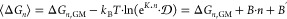
14where *B* and *B*′ are fitting parameters. The above equation has the same format as eq 10, and therefore we can assume that *B* and *B*′ parameters already contain corrections for both actual monomer pressure versus reference pressure and other low-lying minima. Hence, the *B* parameter is manipulated to obtain an energy barrier of 5 kcal/mol, which is used as our definition of onset of nucleation. The manipulation of *B*′ parameter is irrelevant as it would just shift the energy profile along the energy axis and the nucleation rate depends on the difference between the minimum and the maximum which is unaffected in such a shift.

To sum up, combining eqs 10, 11, and 14, we scale our QC data to obtain Gibbs free energy profiles at a condition where they have an energy barrier of approximately 5 kcal/mol
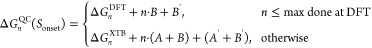
15This approach allows us to estimate the positions of prenucleation and critical clusters, which is the aim of this project.

## 4 Results

### 4.1 Configurational Sampling

We performed CS for ion^±^(H_2_O)_*n*_ and ion^±^(BuOH)_*n*_ at the XTB (GFN1-xTB) level of theory followed by DFT (ωB97X-D/6-31++G(d,p)) calculations for a representative sample of each cluster as described in Methodology (section 3). Up to 5 free-energy lowest structures from both methods were further analyzed to understand the molecular mechanism behind ion-induced nucleation (these structures are provided in SI-2).

**Figure 3:**
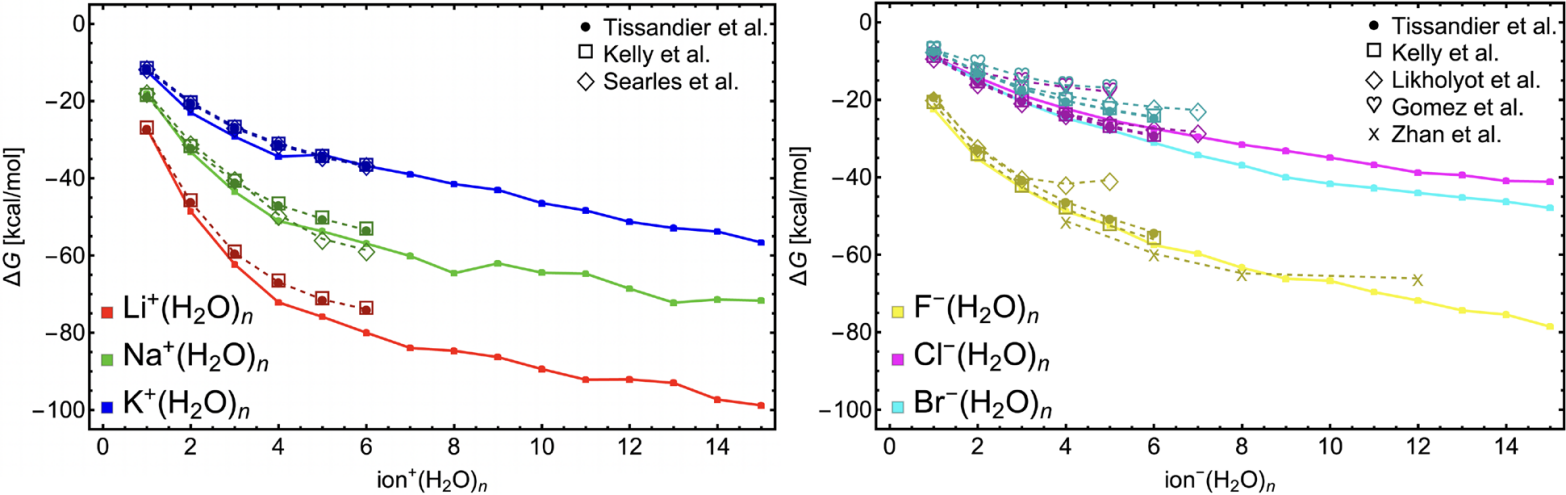
Comparison of standard Gibbs free energies of formation between this study and several other studies for all ion–water clusters.^38−44^ All data from this study (solid-line connected points) were calculated at the ωB97X-D/6-31++G(d,p) level of theory.

Figure 3 shows the calculated standard (i.e., *T* = 298.15 K and *p*^ref^ = 1 atm) Gibbs free energies of formation (Δ*G*_*n*_^DFT^) for all ion^±^(H_2_O)_1–15_ global minimum structures (the numerical values can be found in SI-3). For cations, the energy steeply drops with few (4–5) water molecules condensed and afterward decreases moderately. For the anions, a moderate energy decrease is observed already at smaller sizes. The energy gain is larger for small ions compared to the large ones, in line with the predictions of the CKT theory. However, the energy decrease predicted by the QC calculations is larger than CKT respective predictions. For each ion, as the distance between the core ion and vapor molecules increases, the interaction between the ion and vapor molecules weakens and the energy profile is more and more dependent on the vapor–vapor interaction. Interestingly, a similar energy profile can be observed for Na^+^(H_2_O)_1–15_ and F^–^(H_2_O)_1–15_ as both core ions have similar radius (see Table 1). The Cl^–^(H_2_O)_1–15_ does not seem to follow the trend of the periodic table, which is due to the ion hydration mechanism (see described later in section 4.3, Molecular Mechanism).

Although our CS is not thorough (especially for large clusters) and QC methodology is not as accurate as those used in some other studies, the free-energy profiles for water addition mostly replicate those reported in the previous publications.^38,39,41−44^ One exception is the Br^–^(H_2_O)_*n*_ profile, where we obtain significantly lower energies than most other studies, which can be due to the small basis set used for bromine atom as well as neglect of relativistic effects. In many cases (e.g., for the Li^+^ and F^–^ hydration), the clusters become more stable at large cluster sizes than in the previous studies. Although the ωB97X-D is a size-extensive method, the basis set superposition errors of 6-31++G(d,p) caused by its small size most likely grow with systems size, which can be the reason behind this tendency.^49^ Nevertheless, these comparisons support our choice of methodology. Thus, we believe that by using our methodology, the structural properties can be analyzed to open new insight into the molecular mechanism of ion-induced nucleation.

### 4.2 Energy Profiles

#### 4.2.1 Extrapolation to Large Cluster Sizes

The Gibbs free energies for all ions and solvents calculated at XTB and at DFT are numerically and graphically presented in SI-3. Here, we present the combination of these energy sets. Since the first four vapor molecules in ion^±^(vapor)_1–4_ clusters are dominated by the ion–vapor interaction, we do not use them for the extrapolation to larger sizes. Thus, when applying eq 15, we only used ion^±^(H_2_O)_4–15_ and ion^±^(BuOH)_4–8_ as the basis for fitting. The fitted parameters *A* and *A*′ are presented in SI-4.

**Figure 4:**
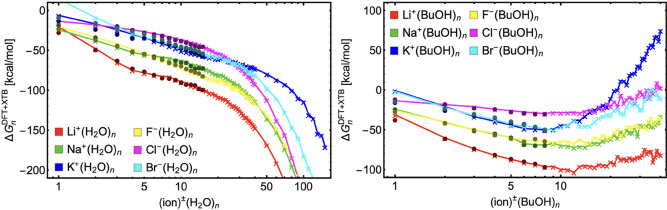
Gibbs free energy profiles of ion^±^(vapor)_*n*_ clusters at standard conditions (i.e., *T* = 298.15 K and *p*_ref_ = 1 atm) calculated at DFT level (dots) and extended with calculations at XTB level of theory (line with crosses).

Figure 4 shows the global free-energy minimum profiles of all ion^±^(vapor)_*n*_ clusters. On the scale the figures have been plotted, all profiles have similar shapes. The corrected XTB energies (represented by lines with crosses) seem to adequately extrapolate the DFT energies (dots). Some deviations between DFT and XTB can be observed for the smallest cluster sizes which were excluded from the fitting. A few remarks on the extrapolated energies for large cluster sizes are in order: First, hydration/butanol solvation converge to different bulk properties for each ion; i.e., the slopes (first derivatives) of different curves differ for the largest clusters. Ideally, they should converge to the same bulk properties as each system will become the same droplet with a negligible effect from the dissolved ion. Nevertheless, we do not correct for this inconsistency as we do not want to introduce any correction based on bulk properties. Additionally, it would be difficult to estimate various bulk properties (e.g., surface tension) as their definitions become sound for larger droplet size (at least 2 nm particles).^13,61^ Second, the ion^±^(BuOH)_*n*_ energies grow for the largest clusters. This would suggest that butanol is a gas in standard conditions, which it obviously is not. This discrepancy is most likely due to the exclusion of other local energy minima (as only the global minimum is assumed) and thus complete neglect of the conformational entropy of the clusters. The global free energy minimum dominates only at very strongly binding (crystal-like) clusters whereas in the case of weakly binding clusters with many degrees of freedom the population of other minima significantly affects the free energy of the system. The conformational entropy also affects the hydration (water solvation), but it is likely to be more significant for butanol solvation due to more degrees of freedom in butanol molecules compared to water molecules.

It is beyond the scope of this work to evaluate the conformational entropy. Therefore, we only correct the energy profiles based on eq 15 to obtain free energies at conditions for which the energy barrier height given by the energy difference of prenucleation and critical cluster corresponds to 5 kcal/mol. In practice, we iteratively modify parameter *B* to obtain new Δ*G*_*n*_^QC^(*S*_onset_) (see eq 15), until the corresponding barrier height is reached. However, the effect of deviations coming from some outliers that appeared after due to inaccurate CS significantly impacted the barrier height. Hence, we use regression to approximate the data by function *f*. The choice of the regression function is not important as long as it can capture the minimum and maximum of the data. We use the form of the CKT equation (eq 4) as it reflects the physical nature behind this process. However, since its individual parameters are physically meaningless at small droplet sizes (CKT uses bulk properties), we fit those parameters to our data. In practice, we iteratively modify parameter *B* to obtain new Δ*G*_*n*_^QC^(*S*_onset_) (see eq 15), which we then model by function *f*:
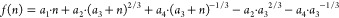
16After fitting the regression parameters *a*_1–4_, we calculate the energy barrier height from the difference between minimum and maximum of modeling function *f*. The iteration of *B* continues until the barrier does not reach 5 kcal/mol. After fitting the regression parameters *a*_1–4_, we calculate the energy barrier height from the difference between minimum and maximum of function *f*. The iteration of *B* continues until the barrier does not reach 5 kcal/mol. The use of function *f* reduces the effect of deviations coming from some outliers that appeared due to inaccurate CS. The function *f* actually has the same form as the CKT equation (eq 4); however, its individual parameters are physically meaningless as we know that CKT predictions typically fail at small droplet sizes. Nevertheless, both CKT parameters and the fitted parameters *B* are shown in SI-4 (the parameter *B*′ is irrelevant in our study). We present and analyze the hydration and the butanol solvation of ions separately in the following sections.

#### 4.2.2 Microhydration of Ions

**Figure 5:**
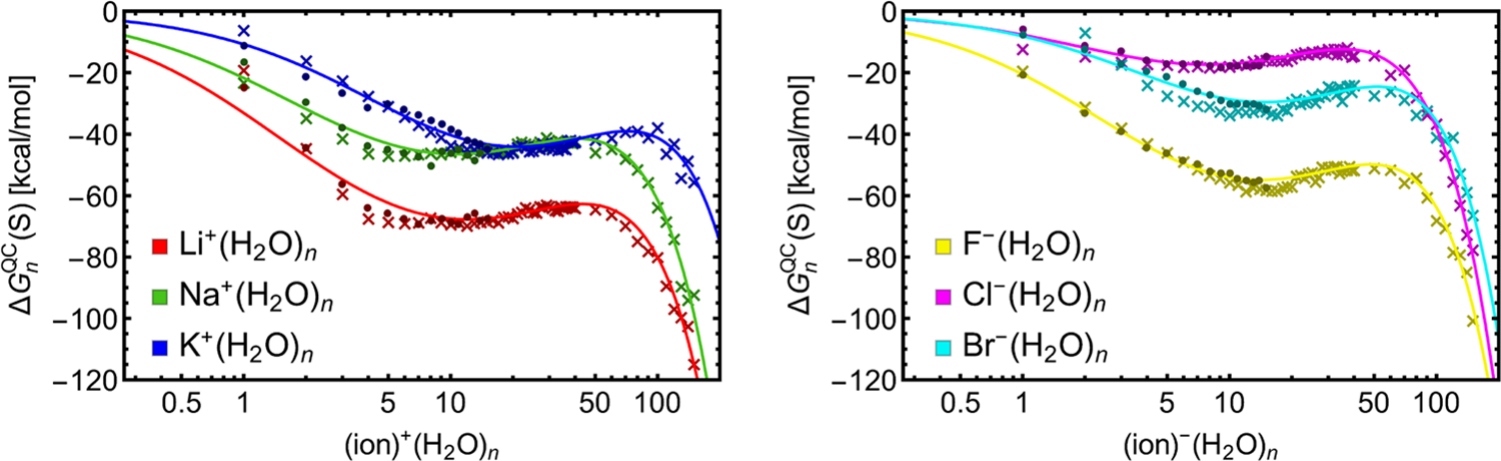
Gibbs free energy profiles of ion^±^(H_2_O)_*n*_ from QC data (i.e., DFT as dots and XTB as crosses) at conditions which approximately correspond to an energy barrier of 5 kcal/mol or equilibrium saturations of *S* = 3.425 for water and *S* = 2.910 for butanol, respectively. The fitted function *f* (eq 16) is presented by a solid line.

Figure 5 shows the free-energy profiles for the ion^±^(H_2_O)_*n*_ systems. The fitted values of *n*_pre_ and *n*_crit_ are presented in Table 4. Li^+^(H_2_O)_*n*_ and Na^+^(H_2_O)_*n*_ reveal similar positions of *n*_pre_ and *n*_crit_. These positions are slightly larger but again similar for F^–^(H_2_O)_*n*_ and Br^–^(H_2_O)_*n*_. (However, keep in mind that the results for systems involving bromine ion are less reliable as explained before.) Due to the ion size and thus different mechanisms of hydration (discussed later), the position of *n*_pre_ and *n*_crit_ for K^+^(H_2_O)_*n*_ is shifted to larger sizes compared to other cations. Cl^–^(H_2_O)_*n*_ exhibits very different behavior. This is impossible to explain from the energy profiles but will be discussed later in the Molecular Mechanism section (section 4.3). Comparing these results to CKT theory predictions (*n*_pre_ ≈ 18 and *n*_crit_ ≈ 100), the QC values of *n*_pre_ and *n*_crit_ are clearly smaller, and as stated before, the ion size and chemical identity have a significant impact on the clustering.

**Table 4:** Number of H_2_O Molecules in the Prenucleation (*n*_pre_) and Critical (*n*_crit_) Cluster Obtained from QC Using the Fitted Function *f* (eq 16) in Figure 5 and from CKT

	system	*n*_pre_	*n*_crit_	system	*n*_pre_	*n*_crit_
QC	Li^+^(H_2_O)_*n*_	∼12	∼43	F^–^(H_2_O)_*n*_	∼14	∼48
	Na^+^(H_2_O)_*n*_	∼11	∼41	Cl^–^(H_2_O)_*n*_	∼8	∼35
	K^+^(H_2_O)_*n*_	∼21	∼71	Br^–^(H_2_O)_*n*_	∼15	∼52
CKT	ion^+^(H_2_O)_*n*_	∼18	∼100	ion^–^(H_2_O)_*n*_	∼18	∼100

#### 4.2.3 Butanol Microsolvation of Ions

**Figure 6:**
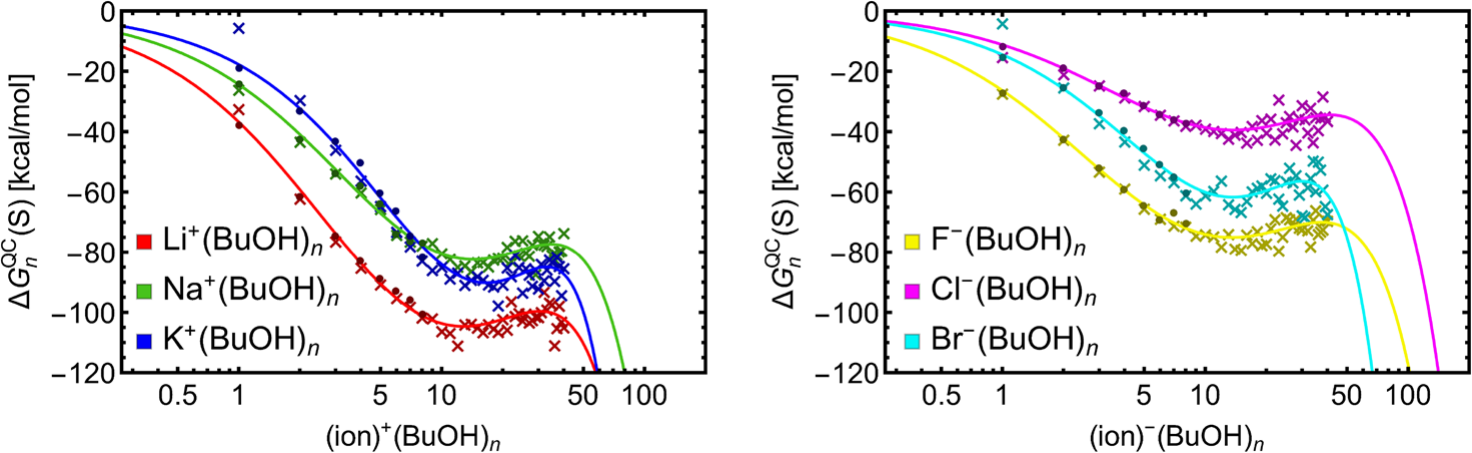
Gibbs free energy profiles of ion^±^(BuOH)_*n*_ from QC data (i.e., DFT as dots and XTB as crosses) at conditions which approximately correspond to an energy barrier of 5 kcal/mol. The fitted function *f* (eq 16) is presented with a solid line.

Figure 6 shows the free-energy profiles for the ion^±^(BuOH)_*n*_ systems. The fitted values of *n*_pre_ and *n*_crit_ are presented in Table 5. The CS of these systems is very complicated, and it can be seen that the ion^±^(BuOH)_20–40_ free energies are burdened with substantial fluctuations from size to size, which is most likely a result of incomplete PES exploration. This consequently affects the accuracy of *n*_pre_ and *n*_crit_ evaluation. We observe a trend in prenucleation clusters for positively charged ions: *n*_pre_(Li^+^(BuOH)_*n*_) < *n*_pre_(Na^+^(BuOH)_*n*_) < *n*_pre_(K^+^(BuOH)_*n*_) but no trend for negatively charged ions. Although there are some slight variations, all ions generally exhibit similar values of *n*_pre_ and *n*_crit_. This means that the mechanism of butanol solvation of ions is different than in the case ion hydration. We further address the different mechanisms in the Molecular mechanism section (section 4.3). (Again, keep in mind that the results for systems involving the bromine atom are not accurate as explained before.) Comparing these results to CKT theory predictions (*n*_pre_ ≈ 9 and *n*_crit_ ≈ 67), the values of *n*_pre_ and *n*_crit_ are closer for butanol clustering than for water clustering.

**Table 5:** Number of H_2_O Molecules in the Prenucleation (*n*_pre_) and Critical (*n*_crit_) Cluster Obtained from Function *f* in Figure 6

	system	*n*_pre_	*n*_crit_	system	*n*_pre_	*n*_crit_
QC	Li^+^(BuOH)_*n*_	∼13	∼31	F^–^(BuOH)_*n*_	∼14	∼37
	Na^+^(BuOH)_*n*_	∼14	∼35	Cl^–^(BuOH)_*n*_	∼14	∼41
	K^+^(BuOH)_*n*_	∼17	∼33	Br^–^(BuOH)_*n*_	∼14	∼30
CKT	ion^+^(H_2_O)_*n*_	∼9	∼67	ion^–^(H_2_O)_*n*_	∼9	∼67

To summarize, we observe from QC that the hydration is strongly dependent on the ion size and specific chemistry. These effects are less pronounced in butanol solvation compared to water solvation. The ion charge sign plays an important role in the hydration/solvation energy. The differences in vapor properties affect the solvation free-energy profiles but the sizes of prenucleation cluster *n*_pre_ and critical cluster *n*_crit_ are affected less compared to CKT. The nucleation barrier, predicted by the CKT theory, is mostly governed by the surface tension σ and molecular volume *V*_m_. This is because the work needed to create a new surface is directly proportional to σ and *r*_*n*_^2^. The ion radius *r*_0_ does not affect the barrier height, but it affects the entire vertical position of the free energy profile. For instance, Tauber et al.^17^ suggested using an effective excluded volume of solvation molecules to obtain results in line with experiments. Hence by modifying *V*_m_ and *r*_0_ values, CKT can provide results better corresponding to experiments or even our QC results. We show in the SI-4 that the parameters of function *f* would correspond to unrealistic values, showing that CKT cannot describe the ion-induced nucleation of systems with small critical droplet size (<2 nm). The reason behind this is that using bulk properties (e.g., surface tension) is rather meaningless in the case of few molecules. Further, we visually examine the structures given by QC calculations to get a new insight into the molecular mechanism behind ion-induced nucleation.

### 4.3 Molecular Mechanism of Nucleation

The first steps of ion-induced nucleation can be qualitatively described with the theory of solvation, which interprets the explicit interaction of solvent and solute molecules. The theory predicts that the solvent molecules organize themselves in layers around the solute. The first layer of solvent molecules interacts directly with the solute. The second solvation layer interacts with the solvent molecules of the first layer and so on.

Ion-induced nucleation/ion solvation represents a transition of the ion from “vacuum” to a medium with a higher dielectric constant (see Table 1). From molecular point of view, the vapor–vapor interaction is important as it, for instance, affects the saturation vapor pressure and thus the vapor concentration required to observe any nucleation. A dense network of hydrogen bonds in a hydrated cluster leads to a lower saturation ratio required to obtain the same energy barrier as in the case of butanol. We mainly focus on the Coulombic (charge-permanent dipole, charge-induced dipole, etc.) ion–vapor interaction as it determines the formation mechanism of the first few solvation layers and the sizes of prenucleation and critical clusters. The main difference in vapor dielectric constants is caused by the different size of the vapor molecules and by their dipole moment and polarizability properties (see Table 6). Water has a greater dipole moment than butanol, and therefore the interaction between the core ion and H_2_O molecules is stronger. However, BuOH has a greater polarizability and therefore can more effectively redistribute electron density according to present ion charge. This can have an important effect on the molecular mechanism of ion solvation, which we explore in the following two subsections.

**Table 6:** Dipole Moment and Polarizability of the Condensing Vapor Molecules and of the Ions^a^

	μ_0_ [D]	α [Å^3^]
H_2_O	1.855	1.02
BuOH	1.66	7.88
Li^+^	0.0	0.01
Na^+^	0.0	0.06
K^+^	0.0	0.74
F^–^	0.0	0.79
Cl^–^	0.0	2.34
Br^–^	0.0	4.00

a The data were obtained at DFT (ωB97X-D/6-31++G(d,p)) level of theory.

#### 4.3.1 Microhydration of Ions

**Figure 7:**
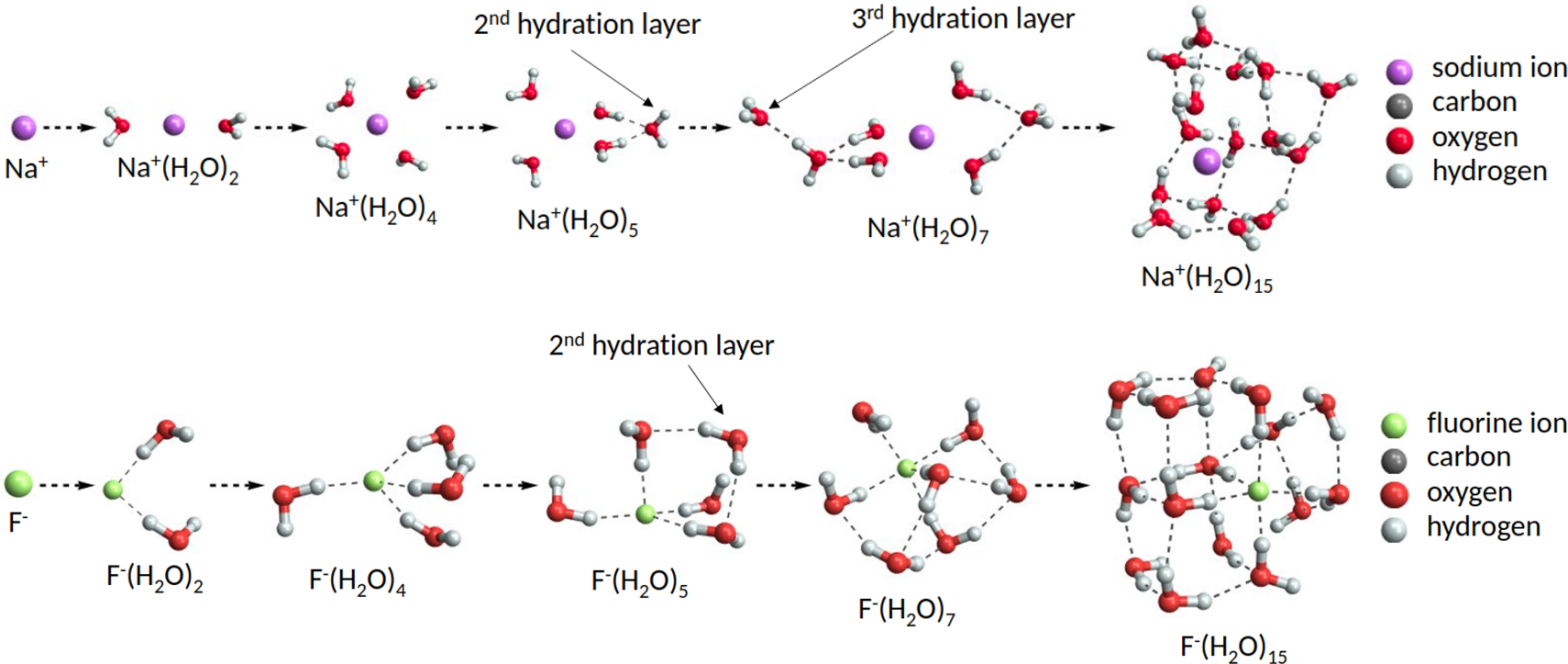
Global minimum structures of the (Na^+^/F^–^)(H_2_O)_0–15_ clusters obtained at the DFT level. Hydrogen bonds are marked with dashed lines.

Na^+^ and F^–^ have very similar diameters, and therefore, we discuss their hydration first. Figure 7 shows the global free-energy minimum structures of several Na^+^(H_2_O)_0–15_ and F^–^(H_2_O)_0–15_ clusters at the DFT level. The first four condensing water molecules interact directly with the core ion. The Na^+^ ion interacts with the oxygen atom (with negative partial charge) of a water molecule, whereas the negatively charged F^–^ ion forms hydrogen bonds with the hydrogen atom (with positive partial charge) of the water molecules. The water molecules in the Na^+^(H_2_O)_2,4_ clusters are oriented in such a way that the distance between them is maximized, as opposed to water molecules in the F^–^(H_2_O)_2,4_ clusters which are oriented so that they also interact with other nearby water molecules. For example, three water molecules in the F^–^(H_2_O)_4_ cluster are oriented so that the hydrogen atom which is not interacting with the F^–^ ion is facing the oxygen atom of the nearest water molecule. This does not result in hydrogen bonding between water molecules as their formation is only observed after the addition of the fifth water molecule to the cluster. Consequently, the “free” hydrogen atom, which is not interacting with the negatively charged core, can interact with other water molecules in the cluster. The resulting F^–^(H_2_O)_2,4_ clusters are asymmetric as opposed to the symmetric Na^+^(H_2_O)_2,4_ clusters. The geometries of Na^+^(H_2_O)_2,4,5_ and F^–^(H_2_O)_3–5_ clusters are similar compared to those found in other computational studies.^62,63^

The driving forces in the formation of the second and third hydration layers are vapor–vapor interactions, ion polarizability, and the ability of the system to redistribute the ion charge. In both cases, the layer starts to form as the newly added water molecules form hydrogen bonds with the water molecules of the pre-existing hydration layer. The second hydration layer starts with the addition of the fifth water molecule in both Na^+^ and F^–^ ion cases. In the Na^+^(H_2_O)_*n*_ system, the third layer starts to form approximately already with the seventh water molecule addition. Nevertheless, the first layer is still subsaturated even for the (Na^+^/F^–^)(H_2_O)_15_ clusters. However, for the F^–^(H_2_O)_*n*_ system, we did not observe formation of the third layer even after the 15th added water molecule. Finally, the structures of the Na^+^(H_2_O)_15_ and F^–^(H_2_O)_15_ clusters show that with increasing number of water molecules in the cluster, the cluster starts to have a rather rectangular-like shape, which gets more spherical when more water molecules are added. Here, we note that the simulated systems ion^±^(H_2_O)_10–15_ contain already too many degrees of freedom and our configurational sampling could have missed some lowest free-energy minima.

The other positive ions, Li^+^ and K^+^, form clusters with water similarly to the Na^+^ ion (cluster geometries are shown in SI-5). The first hydration layer is formed as 1–4 water molecules condense onto the core ion. The water molecules are oriented similarly as in the Na^+^(H_2_O)_1–4_ system; the oxygen atoms are facing the core ion while the hydrogen atoms are pointing out of the cluster. The second layer is likewise formed with the fifth added water molecule. The third layer starts to form at 7 added molecules for Li^+^ ion whereas the formation of the third layer is not observed for K^+^ ion even with 15 added water molecules. This is partly explained by the larger size of K^+^ ion (see Table 1) and the large polarizability of K^+^ (comparable to F^–^; see Table 6), meaning that K^+^ can accommodate more ions to the first layer than the smaller ions. For example, the K^+^(H_2_O)_15_ cluster already has five water molecules in the first hydration layer as opposed to four in the (Li/Na)^+^(H_2_O)_4–15_ clusters. As more ions fit to the proximity of K^+^ ion (i.e., being in the first and second layer) at these cluster sizes, the prenucleation *n*_pre_ and critical cluster *n*_crit_ sizes are also larger.

The other negative ions, Cl^–^ and Br^–^, show a different mechanism of water addition compared to the F^–^ ion (cluster geometries of several Cl^–^(H_2_O)_0–15_ and Br^–^(H_2_O)_0–15_ are also shown in SI-5). Figure 7 shows that the F^–^ ion is slowly getting hydrated from all sides. However, Cl^–^ and Br^–^ ions are only hydrated from one side of the ion. In other words, these ions travel to the surface of the cluster. Such an observation has been seen in molecular dynamics simulations by Caleman et al., who showed that Cl^–^, Br^–^, and I^–^ ions travel from the center of a water droplet to its surface due to water–water interaction being more favorable when the ion is at the surface as opposed to it being fully hydrated.^64^ Cl^–^ and Br^–^ are larger, contain more electrons, and are more polarizable than the other studied ions (see Table 6). As Cl^–^ and Br^–^ undergo hydration, their respective electron clouds are polarized by the first 1–4 added water molecules so that a great part of the electronic density is shifted to the side where the water molecules reside. Conversely, the electronic density on the side that is free of water molecules is so low that all additional water vapor molecules prefer to hydrate on the side where they can form hydrogen bonds with the redistributed charge. The affinity of water molecules to form hydrogen bonds with the negatively charged core ion and other nearby water molecules allows the formation of cluster geometries that contain 5–6, as opposed to 4, water molecules in the first solvation layer.

**Figure 8:**
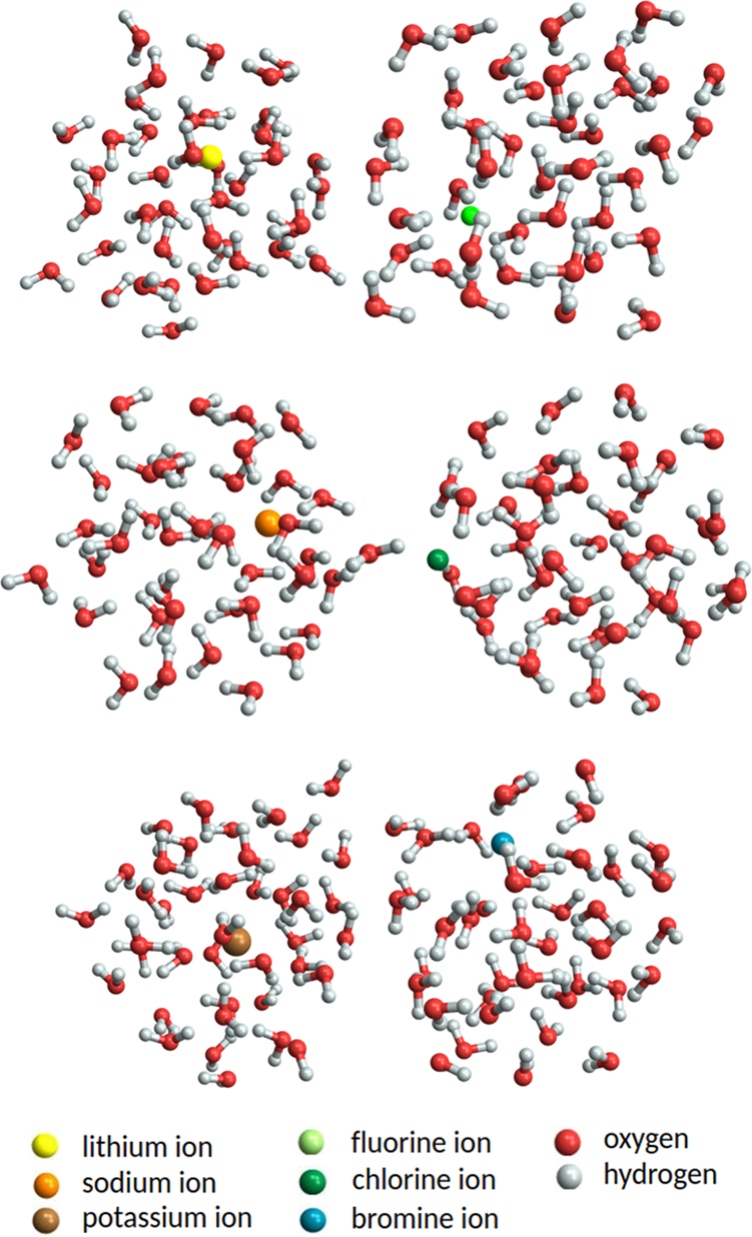
Global free-energy minimum structures of ion^±^(H_2_O)_40_ obtained at the XTB level of theory. The hydrogen bonds are not visualized for better readability.

Figure 8 shows the global free-energy minimum structures of ion^±^(H_2_O)_40_ clusters obtained at the XTB level of theory. All clusters are roughly spherical in shape without any cohesive internal structure, compared to the rectangular-like geometry of the (Na^+^/F^–^)(H_2_O)_15_ clusters in Figure 7. All positive ions (i.e., Li^+^, Na^+^, and K^+^) and the F^–^ ion are hydrated by the surrounding water molecules and stay inside the cluster (not necessary exactly at the center). Conversely, Cl^–^ ion is hydrated only from one side, leaving the other side completely free of water molecules. In our study, the Br^–^ ion is close to the surface but not out of the cluster. Hence, we suspect that either the computational level (XTB) or configurational sampling fails to describe the physics of Br^–^ ion hydration accurately at these large sizes. These findings are in agreement with a study of Tobias and Jungwirth, who showed through molecular dynamics (MD) simulations that small ions (Na^+^, F^–^) are located at the center of an ion–water cluster while larger polarizable ions (Br^–^, I^–^) tend to be located at the air/water interface.^65^ In addition, the classical Onsager–Samaras model predicts that an ion is repelled from the air/water interface by its image charge on the air side.^66^ In the context of spherical ionic clusters, this means that the ion is located at the center of the cluster. However, as this model does not account for the ion polarizability it can only predict the behavior of small, nonpolarizable ions.

**Figure 9:**
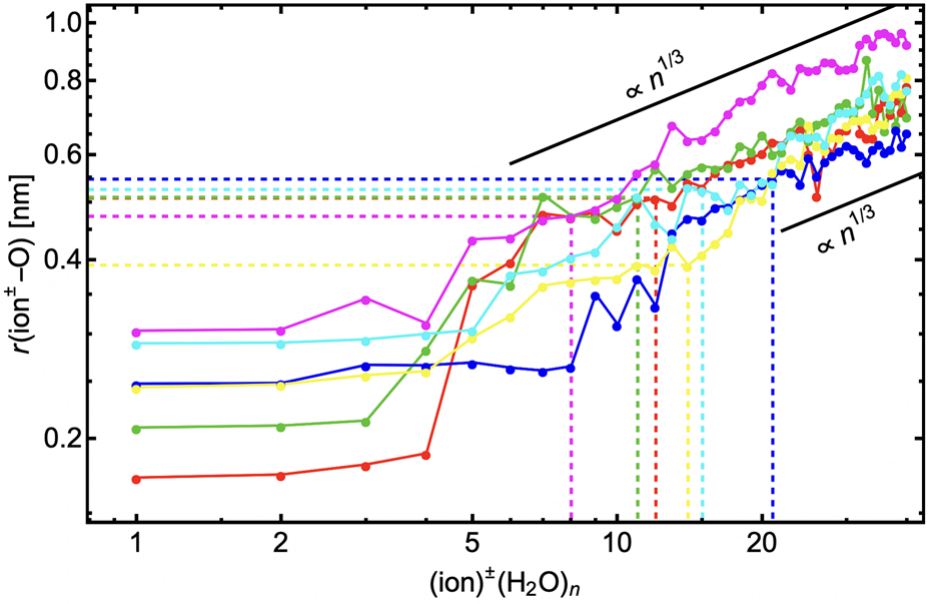
Distance between the core ion and the outermost oxygen atom (of H_2_O) averaged over the five energetically lowest structures at the XTB level of theory. The number of H_2_O molecules in the prenucleation cluster for each core ion is shown with dashed line. The black lines visualize the slope ∝ *n*^1/3^. Note the logarithmic axes.

Finally, we also analyzed the geometrical properties of the hydration. The cluster geometries for ion^±^(H_2_O)_1–15_ are in most cases similar between XTB and DFT levels of theory (see comparison in SI-6). Since the calculations at the XTB level cover a wider range of clusters, we used these geometries to calculate the distance between the core ion and the (oxygen of the) outermost water molecule (shown in Figure 9). The *r*(ion^±^–O) is roughly constant during the formation of the first hydration layer. Afterward, *r*(ion^±^–O) grows as the formation of the second, third, and fourth hydration layers begin to form. The newly added water molecules are sometimes adsorbed to the outermost hydration layer even if the inner hydration layers are not yet fully filled (e.g., see geometries of Na^+^(H_2_O)_5_ and Na^+^(H_2_O)_7_ clusters in Figure 7). As more water molecules are added to the cluster, *r*(ion^±^–O) grows approximately ∝*n*^1/3^. The initial behavior of these curves is another reason why CKT cannot capture the behavior at the small droplet sizes. Figure 9 also shows that the number of water molecules in the prenucleation cluster (*n*_pre_) depends, at least partially, on the *r*(ion^±^–O) distance. However, in general, the relationship between the number of water molecules in the prenucleation cluster is governed by the details of the chemical interaction differences. The differences of *n*_pre_ between different core ions cannot be captured at all by the CKT theory, according to which *n*_pre_ ≈ 18 independent of the core ion. Note that *n*_pre_ varies with system conditions (see Figure 2). CKT cannot capture the differences as it assumes bulk properties of the condensing liquid and does not account for individual ion–vapor and vapor–vapor interactions nor for the differences in molecular configurations between different ion clusters that result from ion properties (e.g., polarizability) as mentioned above.

Some studies have proposed a sign-preference for ion-induced nucleation. Nadykto et al.^67^ showed through quantum chemistry calculations that water has a positive sign-preference for monatomic ion hydration. Also, Froyd and Lovejoy^68^ experimentally showed that small negatively charged (HSO_4_^–^)_1_(H_2_SO_4_)_*x*_(H_2_O)_*y*_ clusters have a lower affinity to water (i.e., are less microhydrated) than positively charged (H^+^)_1_(H_2_SO_4_)_*x*_(H_2_O)_*y*_ clusters. On the other hand, Rusanov^69^ showed theoretically that negatively charged particles possess a higher condensation activity, and an experimental study, carried out by Chen and Cheng,^70^ showed that water vapor nucleates more readily onto negatively charged SiO_2_ particles than positively charged SiO_2_ particles. In this work, we show that the free energy gain is more significant in the initial steps of microhydration for positive singly charged ions rather than for negative ones (see Figure 5). However, in both cases we keep the same nucleation barrier height (5 kcal/mol), and thus we do not expect a significant enhancement of the NPF rates at these conditions. From the molecular point of view, we show that in the case of ion^±^(H_2_O)_*n*_ clusters, the size of prenucleation cluster (and the critical cluster) and its dependence on ion properties is difficult to determine. Hence, we conclude that in the case of water vapor, several other factors are present: ion size, ion polarizability, and ion–water interaction leading to different ion-hydration mechanisms. Consequently, a general charge sign-preference of ion-induced nucleation of water vapor was not found even for a simple case of singly charged atoms. Thus, a generalization of sign-preference for more chemically complex ions is probably even more difficult to come by.

#### 4.3.2 Microsolvation of Ions by Butanol

**Figure 10:**
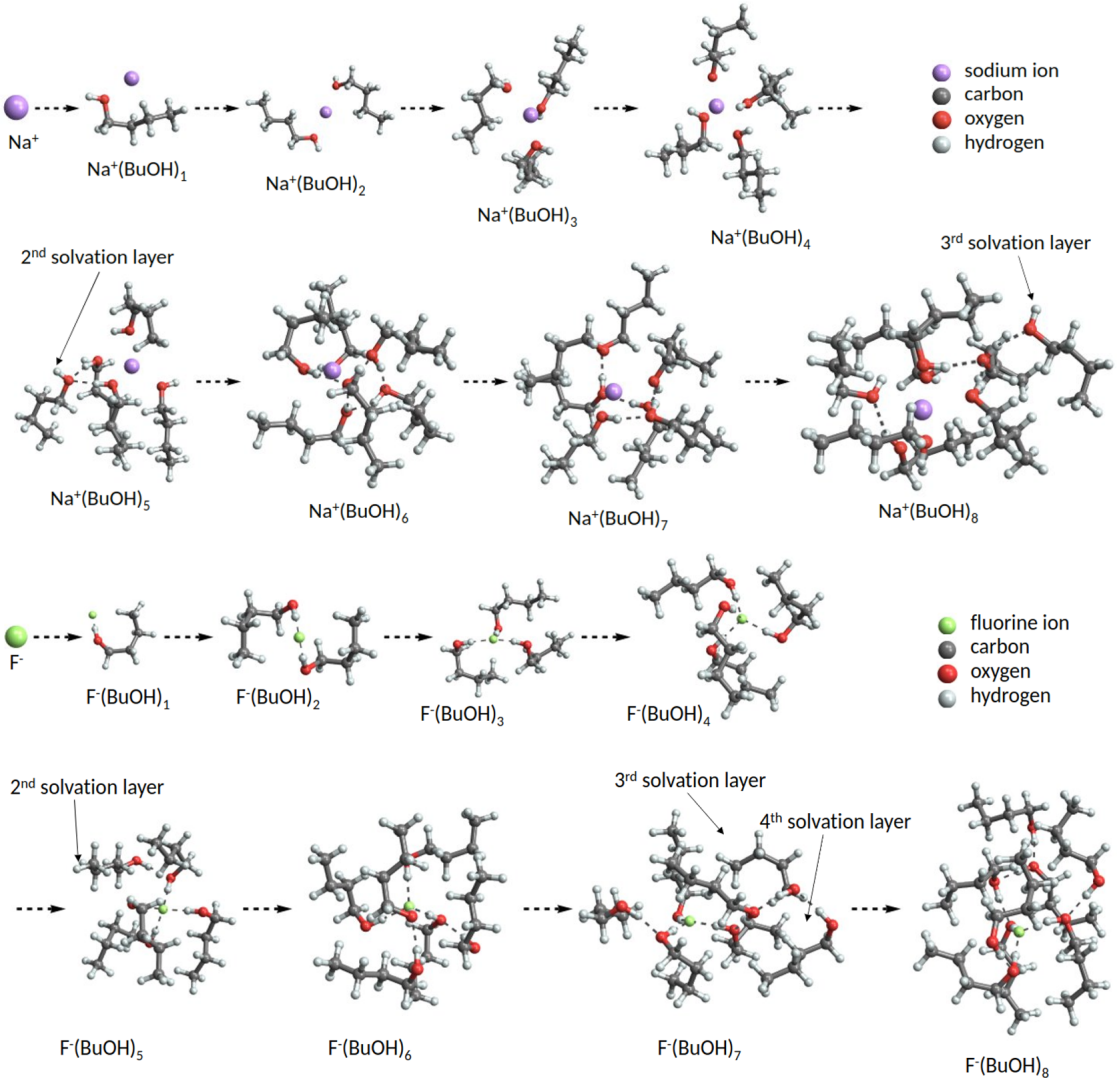
Global free-energy minimum structures of (Na^+^/F^–^)(BuOH)_0–8_ clusters obtained at the DFT level. Hydrogen bonds are marked with dashed lines.

Figure 10 shows global free-energy minimum structures of Na^+^(BuOH)_0–8_ and F^–^(BuOH)_0–8_ clusters at the DFT level. The first four added butanol molecules, which constitute the first solvation layer, interact with the core ion mainly through the polar hydroxyl group. In the case of the negative F^–^ ion, the ion interacts with the hydrogen atom. In the case of positive Na^+^ ion, the ion interacts with the oxygen atom of the hydroxyl group and thus leaves the hydrogen to freely interact with another hydroxyl group. In both cases, the butanol molecules in the first solvation layer are oriented so that the distance between them is maximized. Similar behavior was also observed in all ion^+^(H_2_O)_*n*_ systems, but not in any of the ion^–^(H_2_O)_*n*_ systems (see the SI-5).

The second layer starts to form with the fifth added butanol molecule, which binds itself to the first layer by forming a hydrogen bond with one of the butanol molecules of the first layer. In the case of Na^+^ ion, the newly added butanol molecules may form hydrogen bonds with two butanol molecules of the first solvation layer (e.g., see Na^+^(BuOH)_5_ in Figure 10). This is again possible because the oxygen atoms in the butanol hydroxyl groups of the first solvation layer are oriented toward the core ion leaving the hydrogen atom of the same group pointing outward. The oxygen atom of a newly added butanol molecule can therefore interact with two pre-existing nearby butanol molecules that reside in an inner solvation layer, resulting in two hydrogen bonds. In the case of F^–^ ion, each of the added butanol molecules on the second solvation layer only forms hydrogen bonds with one butanol molecule that resides on the first solvation layer. Similar behavior is observed as more butanol molecules are added to these ions. The third solvation layer starts to form around the eighth butanol molecule addition onto the Na^+^ ion. Similarly, the third solvation layers start to form around the seventh butanol molecule addition to a F^–^ ion. The fourth solvation layer can be seen already at F^–^(BuOH)_7_ which can, however, be an artifact due to inadequate configurational sampling. We tentatively suggest that the free-energy structures of Na^+^(BuOH)_8_ and F^–^(BuOH)_8_ contain 5 and 3 butanol molecules in the first solvation layer, respectively, as opposed to 4. This suggests that ion-induced nucleation involving butanol favors positively charged ions.

**Figure 11:**
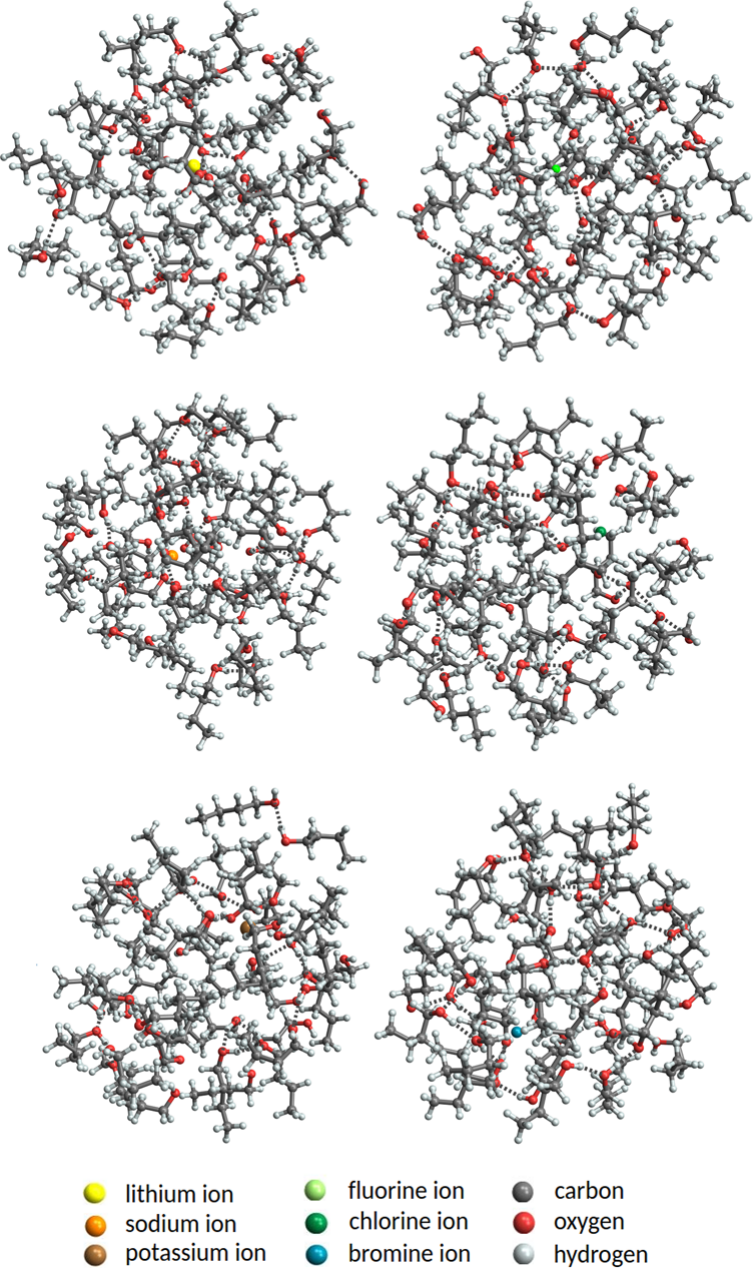
Global free-energy minimum structures of ion^±^(BuOH)_40_ obtained at the XTB level of theory.

Figure 11 shows the global free-energy minimum structures of ion^±^(BuOH)_40_ clusters found at the XTB level of theory. All the small ions (Li^+^, Na^+^, and F^–^) are located roughly at the center of the cluster, whereas all the other ions are shifted off-center. In the case of butanol, all ions are fully solvated as opposed to the ion microhydration. The main interaction mechanism between solvent/vapor molecules is hydrogen bonding. Hence, in all clusters, we can observe alkyl butanol chains extending out from the core ion to the cluster surface. Some of the butanol molecules are not (hydrogen) bound to the inner or the outer solvation layer. For example, at the upper part of the K^+^(BuOH)_40_ cluster picture (see Figure 11), there are two butanol molecules that are bound to each other with a hydrogen bond but are not bound by any hydrogen bond to butanol molecules in the inner layers. Similar chains are also observed inside of this and other clusters. This kind of structure is possible because butanol is highly polarizable and can redistribute electronic density upon introducing an electric field. This gives rise to the creation of induced dipoles within butanol molecules that interact with both the core ion and the surrounding dipoles. This is in contrast to ion^±^(H_2_O)_*n*_ clusters, in which the mutual interaction of water molecules was clearly observed to occur through hydrogen bonds and a dense hydrogen bond network forms.

**Figure 12:**
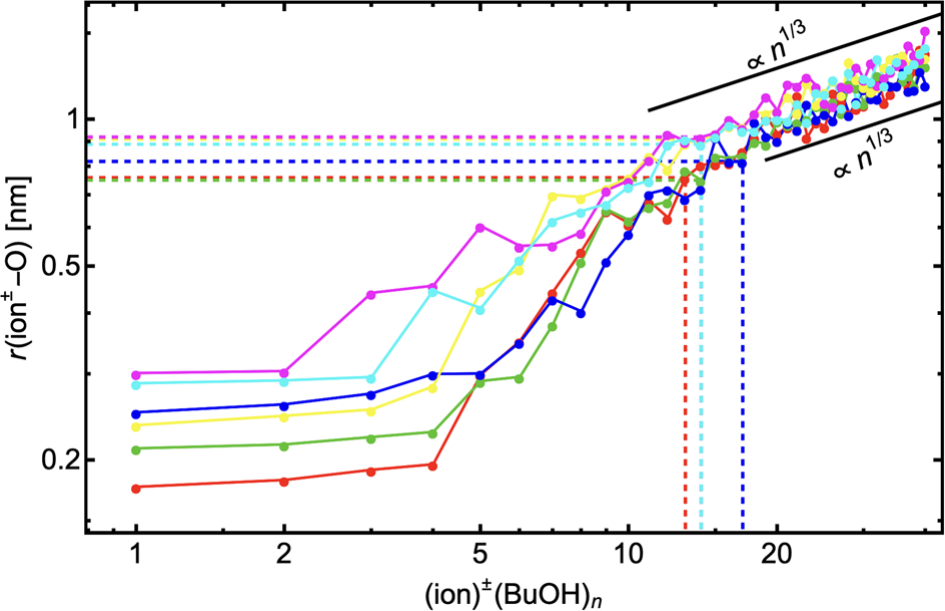
Distance between the core ion and the outermost oxygen atom (of BuOH) averaged over the five energetically lowest structures at the XTB level of theory. The number of BuOH molecules in the prenucleation cluster for each core ion is shown with dashed line. The black lines visualize the slope ∝ *n*^1/3^. Note the logarithmic axes.

Figure 12 shows the distance between the core ion and the outermost oxygen atom in the cluster. This distance stays almost constant during the formation of the first solvation layer for all ion–butanol clusters. This again is not reliably predicted by the CKT theory. As subsequent solvation layers are formed, the distance *r*(ion^±^–O) is roughly proportional to *n*^1/3^ as was also observed in the ion^±^(H_2_O)_*n*_ system. After the first solvation layer has been formed, the *r*(ion^±^–O) grows most rapidly as newly added butanol molecules bind themselves to the outermost solvation layer. This results in the cluster becoming almost spherical in shape. Even the *r*(ion^±^–O) of Cl^–^(BuOH)_*n*_ seems to follow the same trend. Although butanol does initially attach only on one side of the cluster (third BuOH molecule addition), with increasing number of condensed butanol molecules, the first layers are filled and *r*(ion^±^–O) again grows as *r*(ion^±^–O) ∝ *n*^1/3^ as in the case of other ions.

Figure 12 also shows that the distance between the core ion and the outermost oxygen atom is clearly greater for negatively charged ion clusters than for positively charged clusters (even when ion size is taken into account). The variation of *r*(ion^±^–O) is smaller than in the ion^±^(H_2_O)_*n*_ case. Also, the number of BuOH molecules in the prenucleation cluster *n*_pre_ (from QC calculations) varies significantly less than in the ion^±^(H_2_O)_*n*_ case. We compared *r*(ion^±^–O) at *n*_pre_ size for each ion (see horizontal dashed lines), which clearly highlights that for the positively charged ions the prenucleation cluster is located at smaller cluster radii (not necessarily for BuOH molecules). Based on this, we assume that the critical cluster will be located at smaller cluster radii too. The smaller the cluster size, the stronger the ion charge effect. Hence, we expect the positively charged postcritical clusters to grow faster than the negatively charged ones. Therefore, we predict that ion-induced nucleation has a positive sign-preference in the ion^±^(BuOH)_*n*_ systems. This observation is in agreement with our previous study where we also showed positive charge preference of butanol condensing onto a (Na_*x*_Cl_*y*_)^*x*−*y*^ seed.^25^ A positive sign-preference was experimentally observed by Tauber et al.;^17^ they showed that the needed butanol supersaturation to activate positive ions has to be smaller compared to negative ions.

## 5 Conclusions

In this work, we used computational quantum chemistry (QC) and compared it with classical Kelvin–Thomson theory by applying both to the study of heterogeneous nucleation of water (H_2_O) and *n*-butanol (C_4_H_9_OH) onto three positively (Li^+^, Na^+^, and K^+^) and three negatively charged (F^–^, Cl^–^, and Br^–^) monatomic ions. The aim of the study was to determine the sizes of prenucleation and critical clusters and gain molecular insight into the mechanism of ion-induced nucleation. The quantum chemistry calculations consisted of configurational sampling with QC methods that were verified to provide at least qualitatively meaningful results. Quantitative results would require a more thorough configurational sampling. The calculated Δ*G*^DFT^ were extended with Δ*G*^XTB^ to larger size. Moreover, we introduced an approximation to account for the liquid-like behavior of conformational entropy. By applying this approximation, we obtained the positions of both prenucleation and critical clusters for the case where the nucleation barrier equals ∼5 kcal/mol.

Independent of the core ion, the classical Kelvin–Thomson theory predicts n_pre_ ≈ 18 and n_crit_ ≈ 100 for (ion)^±^(H_2_O)_*n*_ clusters and n_pre_ ≈ 9 and n_crit_ ≈ 67 for (ion)^±^(BuOH)_*n*_ clusters. However, our QC calculations show a clear dependence on the core ion. We predicted n_pre_ ≈ 8–21 and n_crit_ ≈ 35–71 for (ion)^±^(H_2_O)_*n*_ clusters. From a molecular point of view, we showed that several different mechanisms are present in these ion–water systems due to variations in ion size, polarizability, etc. Consequently, no general trend in ion-induced nucleation of water based on a single ion property (e.g., ion size, ion charge sign, polarizability) was found. On the other hand, for the (ion)^±^(BuOH)_*n*_ systems, we found lower variation of n_pre_ ≈ 13–17 and n_crit_ ≈ 30–41. Overall, the positively charged ions were shown to have smaller critical cluster sizes at the studied conditions. Moreover, after analyzing the molecular clusters visually, we pointed out that positive ions interact with the oxygen atom of butanol molecules leaving the hydroxyl hydrogen “free” for interaction with another butanol molecule. This type of stabilization is not present around negative ions. Therefore, we conclude that butanol nucleates more readily onto positively than negatively charged ions, which is in agreement with experiments by Tauber et al.^17^ Nevertheless, we showed that the distance between the core ion and the outermost molecule stays roughly constant during the filling of the first solvation layer. This is a phenomenon that the classical Kelvin–Thomson theory cannot capture, leading to possible quantitative errors in estimating the size of prenucleation/critical clusters and the height of the nucleation barrier.

This work has proven something that was known already: the classical Kelvin–Thomson theory is not accurate at small droplet size, i.e., when the critical cluster consists only a “handful” of molecules. However, to our knowledge, we were the first to show molecular insight into ion-induced nucleation of water/butanol vapors. We can draw several conclusions for the ion–butanol systems, but the diversity of mechanisms in the ion–water systems warrants future investigations.

## Supporting Information Available

SI-1: Reasoning for QC data extrapolation. SI-2: Structures and thermodynamics properties. SI-3: Gibbs free energy profiles at DFT and XTB. SI-4: The regression of function *f*. SI-5: Graphical visualization of molecular mechanisms. SI-6: Distance between ion and the farthest oxygen. (PDF)
