# Supplementary material for: Heterogeneous Ion-Induced Nucleation of Water and Butanol Vapors Studied via Computational Quantum Chemistry beyond Prenucleation and Critical Cluster Sizes
Source: J Phys Chem A. 2023 Apr 26;127(18):3976–90. doi: 10.1021/acs.jpca.3c00066 (PMC10184119; doi:10.1021/acs.jpca.3c00066)
Supplement: Supplementary file 1 — jp3c00066_si_001.pdf [file jp3c00066_si_001.pdf]

Supporting Information:

Heterogeneous Ion-Induced Nucleation of Water and Butanol Vapors Studied via Computational Quantum Chemistry Beyond Prenucleation and Critical Cluster Sizes

Antti Toropainen,[*,†,¶] Juha Kangasluoma,[†] Hanna Vehkamäki,[†] and Jakub Kubečka[‡,¶]

†*University of Helsinki, Institute for Atmospheric and Earth System Research/Physics, Faculty of Science, P.O. Box 64, Helsinki, 00140, Finland*

‡*Aarhus University, Department of Chemistry, Langelandsgade 140, Aarhus, 8000, Denmark*

¶*Contributed equally to this work*

E-mail: antti.toropainen@helsinki.fi

Contents

- SI-1: Reasoning for the QC data extrapolation
- SI-2: Structures and thermodynamics properties
- SI-3: Gibbs free energy profiles at DFT and XTB
- SI-4: The regression of function f
- SI-5: Graphical visualisation of molecular mechanisms
- SI-6: Distance between ion and the farthest oxygen

1 SI-1: Reasoning for QC data extrapolation

Figure S1 shows the difference in the Gibbs free energy of formation between the DFT and XTB levels of theory for all $\mathrm{ion}^{\pm}(\mathrm{BuOH})_{1-8}$ clusters. Now, let's define:

$$\Delta\Delta G = \Delta G_{\mathrm{DFT}}(\mathrm{ion}^{\pm}(\mathrm{BuOH})_{\mathrm{n}}) - \Delta \mathrm{G}_{\mathrm{XTB}}(\mathrm{ion}^{\pm}(\mathrm{BuOH})_{\mathrm{n}}) \tag{1}$$

We see that for small clusters $\Delta\Delta G > 0$ of Li^+, K^+ and Br^- and $\Delta\Delta G < 0$ for Na^+, F^- and Cl^-. In addition, $\Delta\Delta G$ is significantly greater in the case of Br^- than other ions. But, most importantly, the $\Delta\Delta G$ seems to have linear behavior for $\mathrm{ion}^{\pm}(\mathrm{BuOH})_{3-8}$. The linearity is perhaps speculative for the large polarizable ions of (K+ and Br-). However, we believe that the eq. 11 is a good first approximation here and is sufficient for a qualitative study of nucleation in this work. A similar argumentation goes for the $\mathrm{ion}^{\pm}(H_2O)_{1-15}$ clusters in Figure S2. We further believe that the difference has a linear behavior even for larger clusters as the number of atoms, bonds, and short-range interactions grows linearly with the number of solvent molecules too, i.e. the error changes linearly.

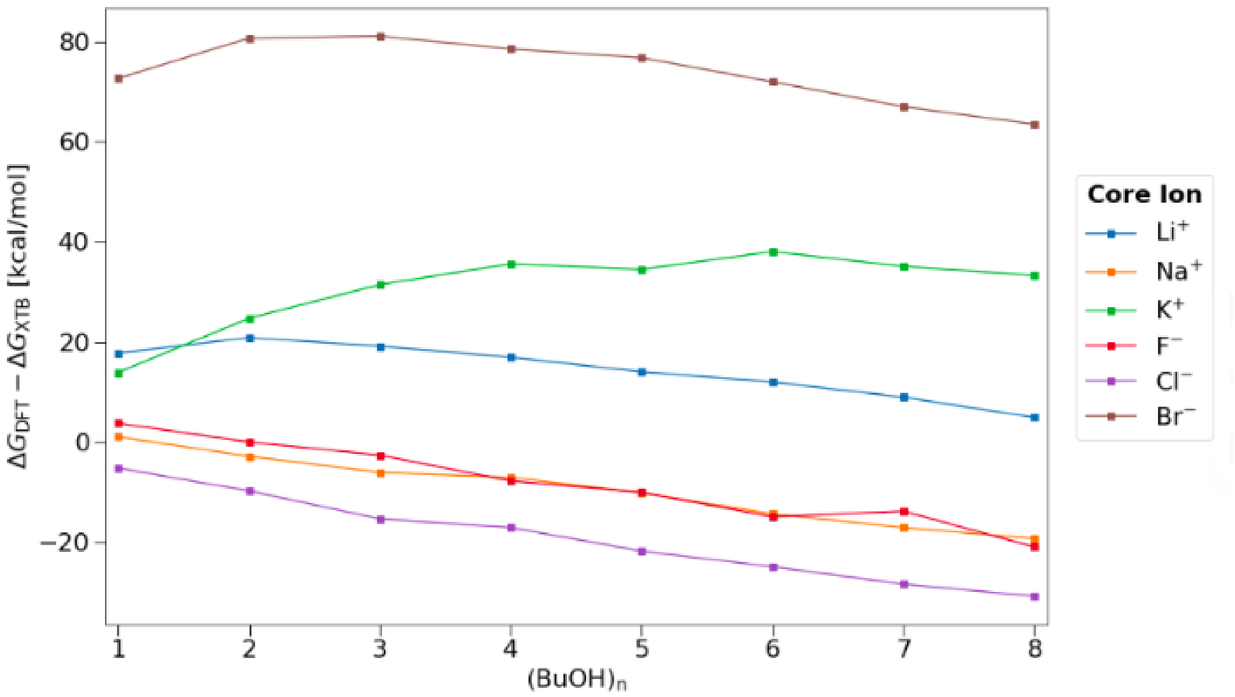

Figure S1: The difference in the lowest binding free energy between the DFT and XTB levels of theory for all $ion^{\pm}(BuOH)_n$ clusters.

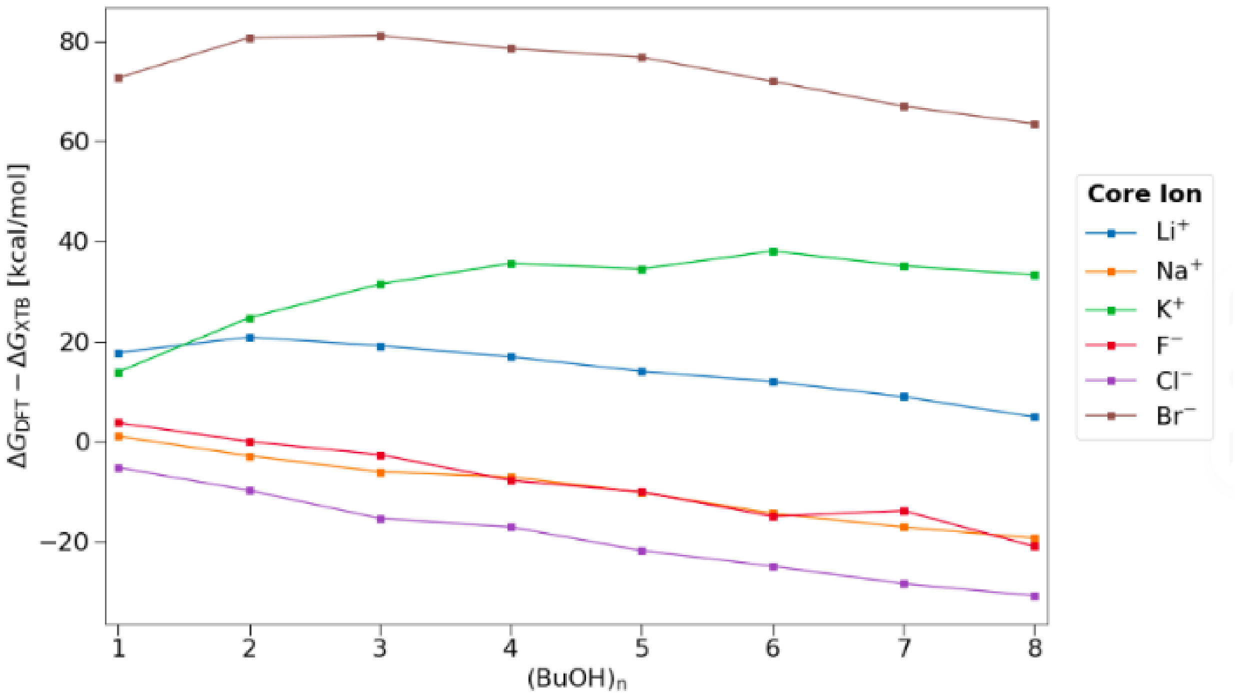

Figure S2: The difference in the lowest binding free energy between the DFT and XTB levels of theory for all $ion^{\pm}(H_2O)_n$ clusters.

2 SI-2: Structures and thermodynamics properties

The cluster thermodynamic properties, as well as structures for (up to) the 5 lowest binding free energy configurations of each cluster, can be downloaded from the GitHub repository:

`https://github.com/kubeckaj/SI/Toropainen_jpca_2023`

3 SI-3: Gibbs free energy profiles at DFT and XTB

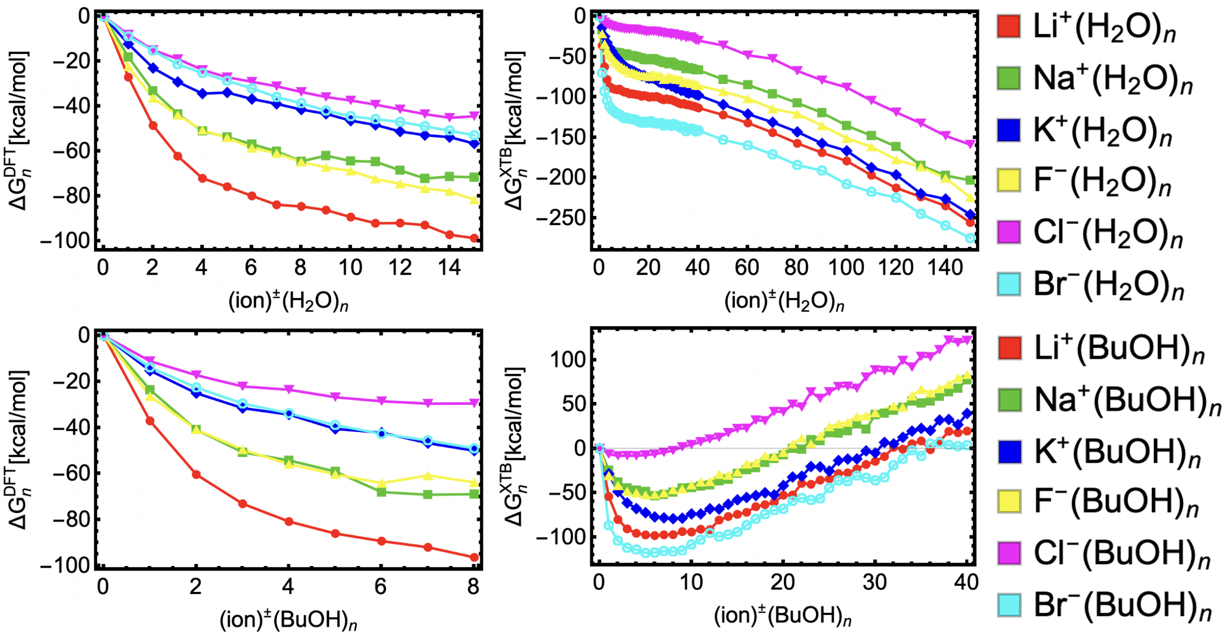

Figure S3: Gibbs free energy profiles of ion$^{\pm}$(H_2O/BuOH)$_n$ clusters at standard conditions (T = 298.15 K and p_{ref} = 1 atm) at the XTB (GFN1-xTB) and DFT (ωB97X-D/6-31++G(d,p)) levels of theory.

4 SI-4: The regression of function f

Table S1: The table of the fitted A and A' parameters, which were used to model the linearly increasing difference between DFT and XTB binding energies. Additionally, the table contains the B parameters, which are optimised to obtain 5 kcal/mol high barrier of the free-energy profile represented by the fitting function f. The parameter B' is irrelevant in this work.

SYSTEM	A	A'	B	B'	$f(n)$
Li^+–H_2O	-1.26	-23.3	2.09	N/A	$-3.1n + 19(0.98n+1)^{2/3} + \frac{201}{\sqrt[3]{0.98n+1}} - 220$
Na^+–H_2O	-1.02	-38.9	1.81	N/A	$-3.1n + 22(0.76n+1)^{2/3} + \frac{167}{\sqrt[3]{0.76n+1}} - 189$
K^+–H_2O	0.45	21.5	0.77	N/A	$-2.3n + 43(0.25n+1)^{2/3} + \frac{213}{\sqrt[3]{0.25n+1}} - 256$
F^-–H_2O	-0.81	-23.8	1.63	N/A	$-3.3n + 33(0.51n+1)^{2/3} + \frac{215}{\sqrt[3]{0.51n+1}} - 248$
Cl^-–H_2O	-1.37	-51.3	1.97	N/A	$-3.1n + 31(0.43n+1)^{2/3} + \frac{115}{\sqrt[3]{0.43n+1}} - 146$
Br^-–H_2O	-0.59	65.9	1.42	N/A	$-3.4n + 60(0.23n+1)^{2/3} + \frac{207}{\sqrt[3]{0.23n+1}} - 267$
Li^+–BuOH	-3.19	-31.1	-0.56	N/A	$-14n + 159(0.35n+1)^{2/3} + \frac{612}{\sqrt[3]{0.35n+1}} - 771$
Na^+–BuOH	-2.84	-48.0	-1.00	N/A	$-12n + 192(0.24n+1)^{2/3} + \frac{601}{\sqrt[3]{0.24n+1}} - 793$
K^+–BuOH	0.20	31.7	-3.99	N/A	$-46n + 1721(0.08n+1)^{2/3} + \frac{2397}{\sqrt[3]{0.08n+1}} - 4117$
F^-–BuOH	-3.14	-48.6	-0.86	N/A	$-7.1n + 81(0.39n+1)^{2/3} + \frac{377}{\sqrt[3]{0.39n+1}} - 458$
Cl^-–BuOH	-2.86	-55.7	-0.97	N/A	$-6.2n + 110(0.21n+1)^{2/3} + \frac{321}{\sqrt[3]{0.21n+1}} - 431$
Br^-–BuOH	-2.54	41.8	-1.40	N/A	$-33n + 973(0.1n+1)^{2/3} + \frac{1447}{\sqrt[3]{0.1n+1}} - 2421$

5 SI-5: Graphical visualisation of molecular mechanisms

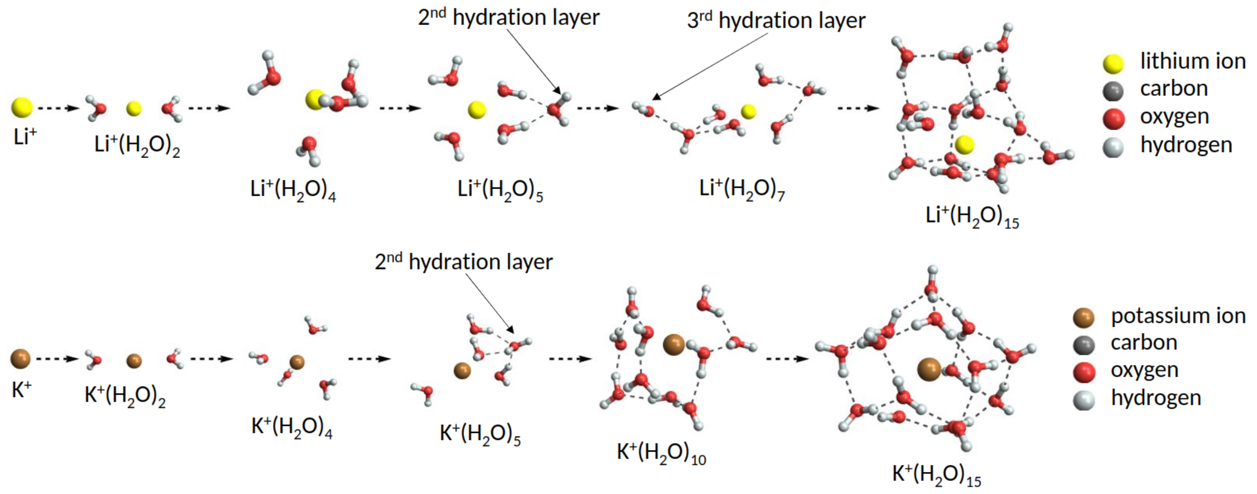

Figure S4: Global minimum structures of the $(Li^+/K^+)(H_2O)_{0-15}$ clusters obtained at the DFT level. Hydrogen bonds are marked with dashed lines.

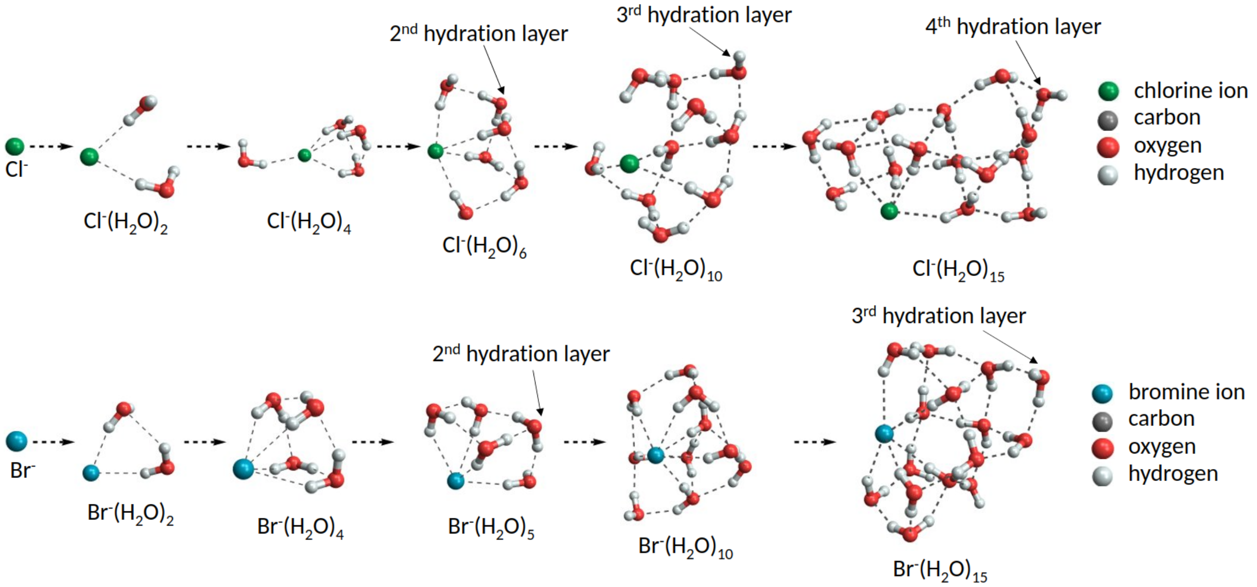

Figure S5: Global minimum structures of the $(Cl^-/Br^-)(H_2O)_{0-15}$ clusters obtained at the DFT level. Hydrogen bonds are marked with dashed lines.

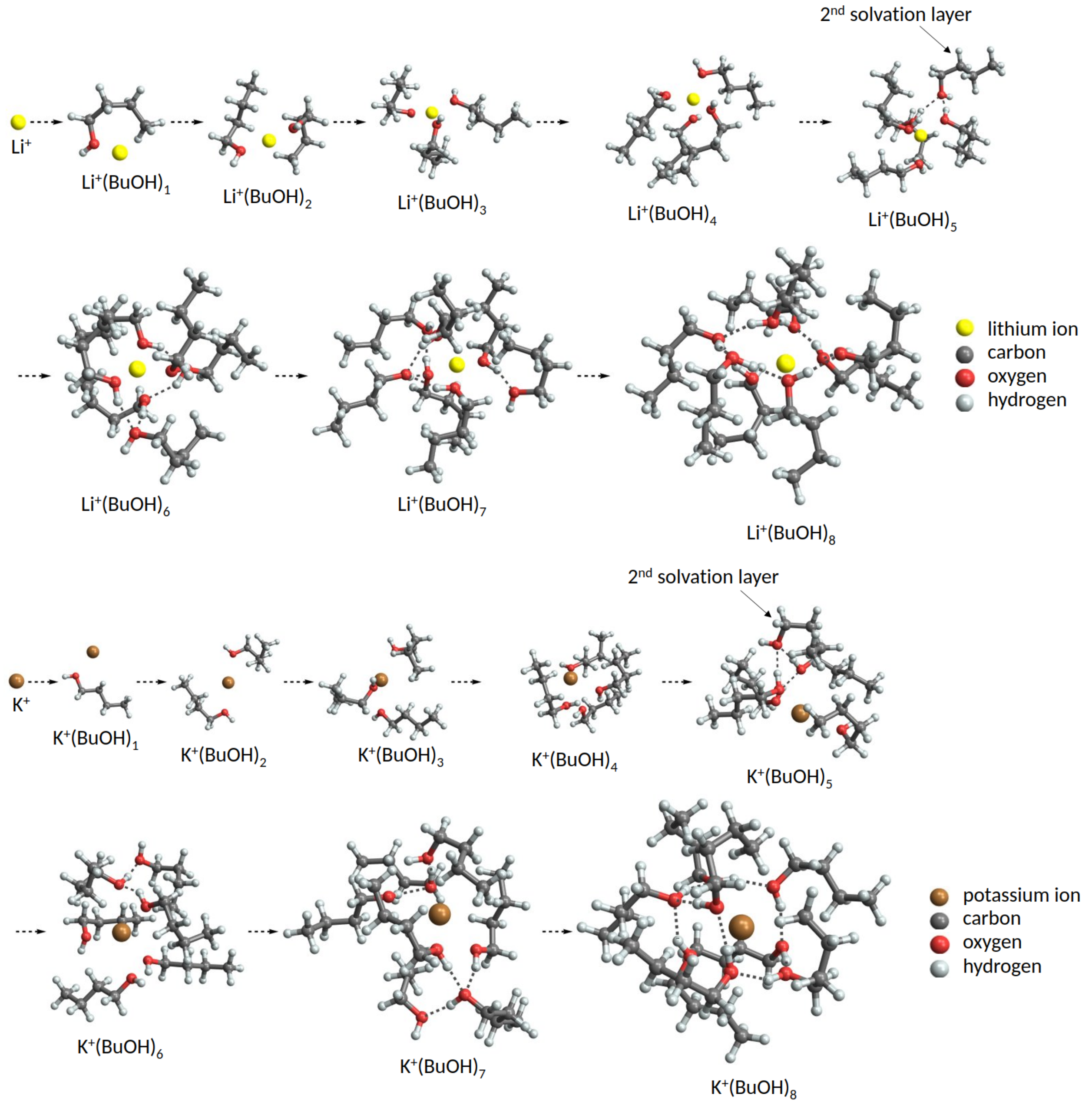

Figure S6: Global minimum structures of the $(Li^+/K^+)(BuOH)_{0-8}$ clusters obtained at the DFT level. Hydrogen bonds are marked with dashed lines.

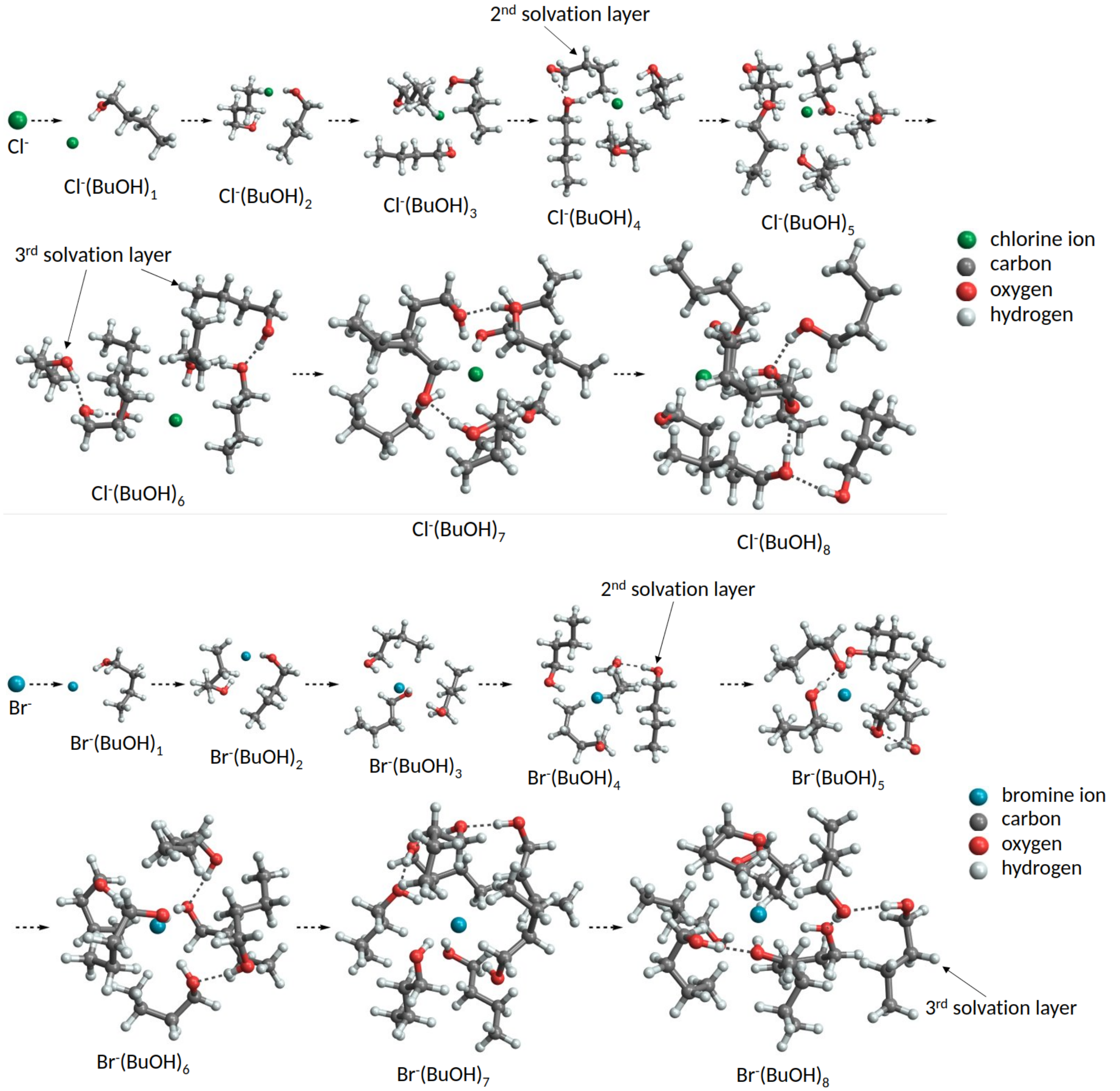

Figure S7: Global minimum structures of the $(Cl^-/Br^-)(BuOH)_{0-8}$ clusters obtained at the DFT level. Hydrogen bonds are marked with dashed lines.

6 SI-6: Distance between ion and the farthest oxygen

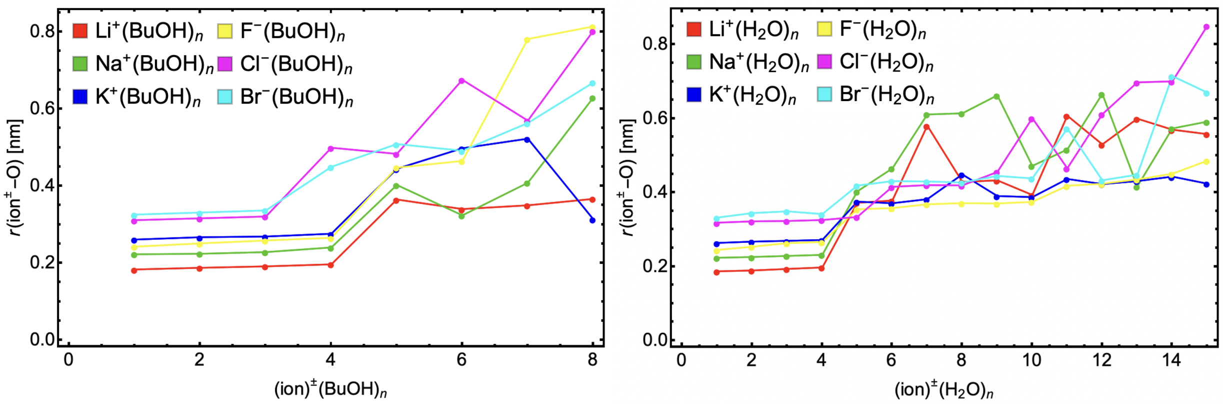

Figure S8: Distance between ion and the most distant oxygen $r(\text{ion}^{+}\text{–O})$ calculated for the global free-energy minimum structures obtained at DFT (ωB97X-D/6-31++G(d,p)) level of theory.
